# Suppression of smooth muscle cell inflammation by myocardin-related transcription factors involves inactivation of TANK-binding kinase 1

**DOI:** 10.1038/s41598-024-63901-3

**Published:** 2024-06-10

**Authors:** Elisabeth Bankell, Li Liu, Jennifer van der Horst, Catarina Rippe, Thomas A. Jepps, Bengt-Olof Nilsson, Karl Swärd

**Affiliations:** 1 Cellular Biomechanics/Vascular Physiology, Department of Experimental Medical Science, BMC D12, Lund University, 22184 Lund, Sweden; 2 Department of Urology, Qingyuan Hospital Affiliated to Guangzhou Medical University, Qingyuan, Guangdong China; 3 Vascular Biology Group, Department of Biomedical Sciences, University of Copenhagen, Blegdamsvej 3, 2200 Copenhagen N, Denmark

**Keywords:** Cell biology, Immunology, Molecular biology, Physiology, Cardiology, Medical research, Molecular medicine, Pathogenesis

## Abstract

Myocardin-related transcription factors (MRTFs: myocardin/*MYOCD*, MRTF-A/*MRTFA*, and MRTF-B/*MRTFB*) suppress production of pro-inflammatory cytokines and chemokines in human smooth muscle cells (SMCs) through sequestration of RelA in the NF-κB complex, but additional mechanisms are likely involved. The cGAS-STING pathway is activated by double-stranded DNA in the cytosolic compartment and acts through TANK-binding kinase 1 (TBK1) to spark inflammation. The present study tested if MRTFs suppress inflammation also by targeting cGAS-STING signaling. Interrogation of a transcriptomic dataset where myocardin was overexpressed using a panel of 56 cGAS-STING cytokines showed the panel to be repressed. Moreover, *MYOCD*, *MRTFA*, and *SRF* associated negatively with the panel in human arteries. RT-qPCR in human bronchial SMCs showed that all MRTFs reduced pro-inflammatory cytokines on the panel. MRTFs diminished phosphorylation of TBK1, while STING phosphorylation was marginally affected. The TBK1 inhibitor amlexanox, but not the STING inhibitor H-151, reduced the anti-inflammatory effect of MRTF-A. Co-immunoprecipitation and proximity ligation assays supported binding between MRTF-A and TBK1 in SMCs. MRTFs thus appear to suppress cellular inflammation in part by acting on the kinase TBK1. This may defend SMCs against pro-inflammatory insults in disease.

## Introduction

Myocardin-related transcription factors (MRTFs: myocardin/*MYOCD*, MRTF-A/*MRTFA*, and MRTF-B/*MRTFB*) are controlled by mechanical stimuli and play a fundamental role in differentiation of smooth muscle cells (SMCs)^1–5^. MRTFs regulate target genes through serum response factor (SRF) which binds one or several genomic CArG-boxes (CC(A/T)_6_GG) to activate genes which regulate contraction, differentiation, and mechanical resilience^4,6–9^. MYOCD is the founding member of the MRTF family^10^, and it is enriched in SMCs where it acts as a driver of differentiation during development^11^. Inducible disruption of the myocardin gene in SMCs of adult mice leads to arterial aneurysms with dissection and rupture^12^, showing that an influence of myocardin in adulthood is critical for arterial patency.

Previous studies supported an anti-inflammatory impact of MRTFs in SMCs. Wang et al. demonstrated a suppressive effect of MRTF-A on *IL1B*, *CXCL2*, and *CCL8* transcription in human pulmonary artery SMCs^13^. This effect involved binding of MRTF-A to RelA/P65 in the NF-κB complex inside nuclei leading to its inhibition. Other studies demonstrated that heterozygous loss of myocardin promotes vascular inflammation and atherosclerosis in dyslipidemic mice^14^, and that myocardin antagonizes NF-κB activity by interfering with RelA/p65 DNA binding^15^. We have confirmed and extended these findings showing that all MRTFs suppress a handful of pro-inflammatory cytokines in human coronary artery SMCs^16^. In some cases (*IL1B*, *CXCL8*) the anti-inflammatory impact of MRTFs appeared to depend on SRF. Moreover, the NF-κB inhibitor dexamethasone only partially prevented MRTF-dependent suppression of inflammation. Together, these findings speak against RelA sequestration as the only mechanism, and additional anti-inflammatory mechanisms of MRTFs may thus exist.

A branch of innate immunity that has received considerable attention in recent years is the cGAS-STING pathway^17–20^. Double-stranded (ds) DNA from viruses, damaged nuclei, or mitochondria is bound by cyclic GMP-AMP synthase (cGAS) in the cytoplasm. This leads to synthesis of the second messenger cGAMP which then diffuses to the endoplasmic reticulum where it activates stimulator of interferon genes (STING). The downstream proteins TANK-binding kinase 1 (TBK1) and IKKε subsequently activate the transcription factors NF-κB and IRF3 to drive synthesis of pro-inflammatory mediators. Recent studies have demonstrated that cGAS-STING signaling is downregulated by the mechano-responsive co-activators YAP and TAZ^21,22^. Importantly, SMC deletion of YAP and TAZ promotes aneurysmal disease^21,23,24^, a pathology characterized by severe arterial inflammation. This is reminiscent myocardin deletion in SMCs which also promotes aneurysmal disease^12^. Notably, our work^23–25^ and that of others^26,27^ indicated that YAP and TAZ are upstream regulators of myocardin. This raises the possibility that myocardin, and indeed all MRTFs, may inhibit cGAS-STING signaling, and that this could represent an additional mechanism beyond RelA sequestration by which MRTFs restrain inflammation. The present study was undertaken to address this hypothesis.

## Materials and methods

### Ethics

Cultured cells used were from commercial vendors who take responsibility for informed consent and use of the cells for experimental purposes. Cells were from anonymous donors and procedures conformed to international treaties and guidelines.

### Bioinformatic analyses

Generation of an RNA-sequencing dataset comparing null- (n = 4), and MYOCD-transduced (n = 4) human coronary artery SMCs is described elsewhere^8,28^. Here we compared fold-changes of cGAS-STING target transcripts with the median fold-change of all other transcripts using Wilcoxon signed-rank test. We also compared the proportion of cGAS-STING targets that were reduced (adjusted p < 0.01) with the proportion of all transcripts that were reduced (adjusted p < 0.01) using Fisher exact test.

RNA-sequencing data generated by the GTEx consortium^29,30^ was downloaded as described^31^. Using this dataset, we correlated *SRF*, *MYOCD*, *MRTFA*, and *MRTFB* versus all other transcripts in each of the three arteries. Mean R-values across arteries were calculated, and the R-values for cGAS-STING target transcripts were extracted and compared against the median R-value of the entire list (Wilcoxon signed-rank test). Individual correlations were subsequently tested using the Spearman method in GraphPad Prism. We also stratified tibial artery data into groups with high (top 10%) and low (bottom 10%) SRF expression and compared SMC transcripts and inflammatory transcripts between the groups. Because the aim to compare top and bottom 10% was pre-specified, we focused on the tibial artery where we expected the power to be best to detect differences due to the large sample size.

### Cell culture

Human coronary artery SMCs (C-017-5C, Gibco) were acquired from Thermo Fisher and propagated in Medium-231 (M231500, Thermo Fisher) with smooth muscle growth supplement (5% SMGS, S00725) and PEST (50 U/ml penicillin and 50 μg/ml streptomycin, Biochrom, A 2212) in a cell culture incubator (5% CO_2_ in air). Bronchial SMCs were obtained from the American Type Culture Collection (PCS-130-011) and cultured in Vascular Cell Basal Medium (PCS-100-030) supplemented with Smooth Muscle Cell Growth Kit (ATCC PCS-100-042) and PEST (50 U/ml penicillin and 50 μg/ml streptomycin, Biochrom, A 2212). Medium was exchanged every second day and cells were re-seeded upon confluence. Experiments were performed in passages 2–12. Similar results were obtained irrespective of cell passage. Cell culture incubators were housed in a professionally ventilated barrier facility, and all cell culture work was done using rigorous sterile technique. Conditioned cell culture media was regularly tested for mycoplasma.

### Overexpression and silencing

Replication-deficient adenoviruses were used for overexpression and silencing. These were obtained from Vector Biolabs. Ad-h-MYOCD (ADV-216227), Ad-h-MKL1/eGFP (MRTF-A, ADV-215499), and Ad-h-MKL2 (MRTF-B, ADV-215500) were used for overexpression. Ad-CMV-Null (#1300) was used as control. Cells were harvested 4 or 8 days after initiating the viral transduction, unless stated otherwise. For 8-day transductions, viruses were added twice, first to a multiplicity of infection (MOI) of 200, and after 4 days, when media were changed, to 400 MOI. Ad-U6-h-MKL1-shRNA (shADV-215497) and Ad-GFP-U6-shRNA (Vector Biolabs, #1122, used as control) were used for silencing (1000 MOI, 4 days). For the time-curve experiment, cells were transduced with MRTF-A or null virus (200 MOI) and harvested at different timepoints (8 h, 24 h, 48 h, 96 h and 192 h). For the dose–response curve, the cells were harvested 96 h after initiating the viral transduction. Cells were transduced with different MOI (10, 30, 100 and 300) of MRTF-A virus. Corresponding MOIs (30–300) of null virus were used as controls, and an average value was used for normalization. For the experiments using PDGF-BB-stimulation, cells were transduced with MRTF-A or null virus (200 MOI) for 6 or 8 days. For the time-curve, dose–response curve, and the PDGF-BB experiments, viral transduction was only performed once.

### RT-qPCR

Cells were washed in cold phosphate-buffered saline (PBS) and lysed in RLT lysis buffer (Qiagen). RNA was isolated and purified using the RNeasy mini kit (Qiagen, #74104) and the QIAcube system (Qiagen) according to the manufacturer’s instructions. RT-qPCR was performed using the QuantiNova SYBR Green RT-PCR Kit (Qiagen, #208156) with the reference dye ROX on a StepOnePlus qPCR cycler (Applied Biosystems) with QuantiTect Primer assays from Qiagen (*IL1A* (QT00001127), *IL1B* (QT00021385), *PLAT* (QT00075761), *AREG* (QT00030772), *EREG* (QT00019194), *CXCL1* (QT00199752), *CXCL3* (QT00015442), *CXCL5* (QT00203686), *CXCL8* (QT00000322), *CCL2* (QT00212730), *LIF* (QT00001442), *MMP14* (QT00001533), *MMP3* (QT00060025), *ACTA2* (QT000088102), *MYH11* (QT00069391), *KCNMB* (QT00080493), *CNN1* (QT00067718), *CAV1* (QT00012607), *CGAS* (QT00056147), *TBK1* (QT00078393), *STING1* (QT00055440), *INFB1* (QT00203763), and *18S* (QT00199367)). Primers for *MRTFA* were produced by Eurofins Genomics (Forward: ATGCCGCCTTTGAAAAGTCCA, Reverse: TCTTCCGTTTGAGATAGTCCTCT), as were primers for *SLMAP* (Forward: ATCTCCGGGAGGAGAAGGAC, Reverse: AATGTCAGTGTCCCGCTCAG). *18S* was used as a reference gene and the Pfaffl method (also known as the 2^−ΔΔCt^ method) was used to calculate fold changes^32^.

### Western blotting

6 well cell culture plates were placed on ice, and culture medium was swiftly decanted. After washing twice in ice cold PBS, 55 µL of Laemmli sample buffer [containing protease inhibitor cocktails (Sigma-Aldrich, P8340-1ML) and phosphatase inhibitor cocktails (Thermo Fisher Scientific, 78420)] was added to each well, and cells were scraped with a rubber policeman. Lysates were transferred to Eppendorf tubes, boiled at 100 °C on a heating block for 5 min and then centrifuged at 16,000×*g* for 15 min*.* Aliquots of the supernatants were assayed for total protein contents using the BioRad DC protein assay (BioRad, #5000112). Protein concentrations of the remaining lysates were adjusted to 1 μg/μl using sample buffer. After addition of 2-mercaptoethanol plus bromophenol blue, and heating to 95 °C for 5 min, 20 µg protein was loaded per well on SDS-PAGE gels (BioRad, #5671084, #5671124). Precision Plus Protein™ Kaleidoscope™ Prestained Protein Standards (Bio Rad, #1610375) were loaded in the lateral lanes, and transferred to the membrane together with target proteins. Gels were run at 200 V using Tris/glycine/SDS buffer (BioRad, #1610732). The Trans-Blot Turbo Transfer System (BioRad) was used for transfer to 0.2 μm nitrocellulose membranes (BioRad, #1704159). Membranes were cut in horizontal strips to allow for blotting of multiple target proteins including loading controls. After washing in Tris-buffered saline with 0.1% Tween (TBST), membranes were blocked with 1% casein/TBS (1:1) (BioRad, #1610782) for 2 h at room temperature, and incubated overnight in sealed bags with primary antibody in blocking solution. The following primary antibodies were used: P-TBK1 (Cell Signaling Technology, #5483), TBK1 (Cell Signaling Technology, #3504, 1:500), P-IKKε (Cell Signaling Technology, #8766S), MYH11 (Abcam, #ab53219), SLMAP (Millipore Sigma, #HPA002357), CAV1 (BD Biosciences, #610407), LDHB (Abcam, # ab264358), Histone H3 (Cell Signaling Technology, #4499S), IL-8 (Cell Signaling Technology, #94407), MCP-1 (Abcam, #ab9669), HSP90 (BD Biosciences, #610418), STING (Cell Signaling Technology, #13647S, 1:1000), and P-STING (Cell Signaling Technology, #50907S, and #19781S). After three washes in TBST with continuous shaking, membranes were incubated with the appropriate secondary antibodies (Cell Signaling Technology, #7076 and #7074) diluted in 1% casein/TBS for 2 h at room temperature. After three additional washes in TBST, bands were developed using SuperSignal West Femto substrate (Thermo Fisher Scientific, #34096). The LI-COR Odyssey Fc instrument (LI-COR Biosciences) was used for imaging and quantification.

Calf intestinal alkaline phosphatase (CIP) (Sigma-Aldrich, #11097075001) was used for phosphatase treatment of western blot samples. Cells were lysed in lysis buffer containing protease inhibitor cocktail and either vehicle or phosphatase inhibitor cocktail. After cell lysis and protein determination, samples were resuspended in CIP buffer (100 mM NaCl, 50 mM Tris–HCl, 10 mM MgCl_2_, 1 mM dithiothreitol, protease inhibitor cocktail, pH was adjusted to 7.9 (25 °C)) to a protein concentration of 1 μg/μl. 1 unit CIP per µg of protein was added to the phosphatase samples for dephosphorylation. The corresponding volume of vehicle was added to the control sample (the sample also containing phosphatase inhibitor cocktail), and both samples were incubated for 45 min at 37 °C. We then added 2-mercaptoethanol and bromophenol blue and adhered to the protocol above for the subsequent steps.

### Pharmacological treatment

Human PDGF-BB (Sigma-Aldrich, #SRP3138-10UG) was dissolved in Milli-Q water and used at 50 ng/ml for 3 or 5 days. Polyinosinic:polycytidylic acid (poly I:C) was dissolved in PBS and used at 30 μg/ml for 3 h or 24 h. The STING inhibitor H-151 (InvivoGen, #inh-h151) was used at 3 µM. We used 1 μg/μl of poly(dA:dT)/LyoVec™ (InvivoGen, #tlrl-patc) double-stranded (ds)DNA as positive control to ascertain that H-151 at this concentration inhibits a response that depends on cGAS-STING. Amlexanox, an inhibitor of TBK1/ IKKε, was used at 50 µM. H-151 and amlexanox were dissolved in DMSO, whereas poly(dA:dT)/LyoVec™ dsDNA was dissolved in sterile endotoxin-free water. DMSO and water were included as vehicles as appropriate. H-151 and amlexanox were included 30 min prior to viral transduction and were present throughout the experiment. LPS (E. coli LPS 0111:B4) was obtained from Sigma-Aldrich, dissolved in PBS, and used at 500 ng/ml. Jasplakinolide (Tocris, #2792), inducing actin polymerization, was dissolved in DMSO and used at 100 nM for 24 h. MnCl_2_ (Sigma-Aldrich) was prepared as a 1 M stock solution in Milli-Q water and used at a final concentration of 1 mM. Because the stock solution of MnCl_2_ could not be titrated to physiological pH without precipitation, we used Milli-Q water where pH was adjusted to be the same as in the MnCl_2_ stock solution as vehicle.

### Co-immunoprecipitation of MRTF-A-binding proteins

A purified antibody against MRTF-A (Bethyl Laboratories, #A302-202A) was immobilized to a column with the AminoLink Plus Coupling Resin from the Pierce Co-Immunoprecipitation kit (Thermo Scientific, #26149). A column containing the inactive Control Resin supplied by the kit was used as a negative control. Human coronary artery SMCs transduced with MRTF-A for 4 and 8 days were washed with cold PBS and lysed in IP Lysis/Wash Buffer. The Control Agarose Resin was used to pre-clear the lysate and ~ 0.5 mg protein was added to each column and incubated overnight with continuous shaking at 4 °C. The resin beads were washed with IP Lysis/Wash Buffer until no proteins were detected in the flow-through. The MRTF-A protein complexes were eluted in 30 μl Elution Buffer and assayed by Dot Blot analysis. 1 μl of the eluates were dotted onto a nitrocellulose membrane, followed by blocking in 1% casein/TBS (1:1) for 2 h in room temperature. The membranes were incubated for 3 days at 4 °C with primary antibodies for total TBK1 (Cell Signaling Technology, #3504, 1:500) and P-TBK1 (Cell Signaling Technology, #5483, 1:500), using MRTF-A (Cell Signaling Technology, #14760, 1:1000) and SRF (Cell Signaling Technology, #5147, 1:500) as positive controls and GAPDH (Merck Millipore, #MAB374, 1:1000) and LDHB (Abcam Biochemicals, #ab264358, 1:1000) as negative controls. For protein visualization, the membranes were incubated for 2 h in room temperature with HRP-conjugated secondary antibodies (1:5000, Cell Signaling, #7076 and #7074). Immunoreactivity was detected using SuperSignal West Femto substrate (Thermo Fisher Scientific, #34096), and images were acquired by the LI-COR Odyssey Fc instrument (LI-COR Biosciences). In the experiment where actin polymerization was induced, the cells were transduced with MRTF-A virus for 4 days and treated with 100 nM Jasplakinolide for the last 24 h.

### Proximity ligation assays

To investigate spatial co-localization of total TKB1 with MRTF-A and P-TKB1 with MRTF-A, we performed proximity ligation assays (PLA) using the Duolink in situ PLA detection kit 563 (Sigma-Aldrich, Denmark) on human coronary artery SMCs. Following 4 days of virus transduction of cells growing on 12-mm coverslips, the cells were fixed in 4% paraformaldehyde in PBS at room temperature for 10 min. Subsequently, the cells were permeabilized in 0.1% Triton X-100 for 5 min at room temperature and then blocked with Duolink blocking solution for 30 min at 37 °C. The cells were incubated overnight at 4 °C with pairs of primary antibodies in Duolink antibody diluent solution. The primary antibodies used were TKB1 (Cell Signaling Technology, #3504, 1:50), P-TBK1 (Cell Signaling Technology, #5483, 1:40), and MRTF-A (Santa Cruz Biotechnology, #sc-390324, 1:50). The following day, cells were incubated with combinations of secondary PLA anti-rabbit PLUS and anti-mouse MINUS probes, followed by hybridization, ligation, and amplification steps. Red punctae, indicative of proteins situated within 40 nm of each other, were visualized using a Zeiss LSM900 laser-scanning confocal microscope. Images were analyzed using ImageJ software using the particle detector tool, and the number of puncta was calculated on a single midcell section. Statistical analyses were performed with a Nested t-test.

### Statistics

**Figure 1:**
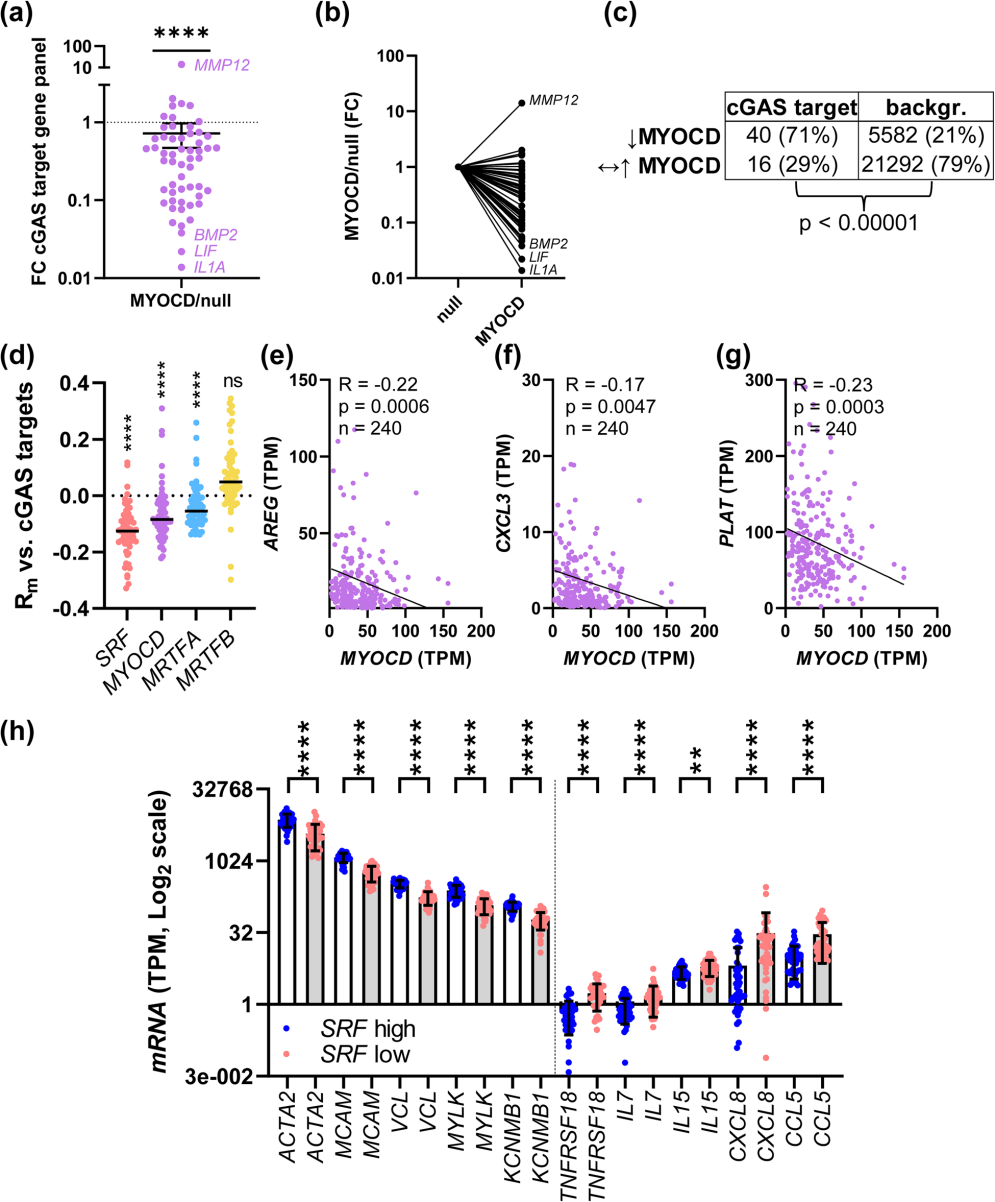
Myocardin is negatively associated with cGAS-STING target genes in cultured human smooth muscle cells and in human arteries. We first interrogated an RNA-sequencing dataset where cells were treated with either null or MYOCD virus for 8 days^28^ with a panel of 56 cGAS-STING target genes from a published study^17^. (**a**) Most cGAS-STING target genes had fold-changes (FC) that were less than 1 and the median FC differed significantly from the median FC of the entire dataset (**a**, one sample Wilcoxon test, n = 4 for null and n = 4 for MYOCD in the underlying RNA-seq). In another approach, cGAS-STING genes were categorized into being either reduced (**b**, adjusted p < 0.01) or unchanged/increased, and the distribution between categories was compared with those in the whole dataset using the Fisher Exact test (**c**, n = 4 null and n = 4 MYOCD samples in underlying RNA-seq). Next, we used RNA-sequencing data from human arteries downloaded from GTExPortal. In panel (**d**), *SRF*, *MYOCD*, *MRTFA*, and *MRTFB* were correlated with all other transcripts in the three arteries in GTEx (n = 240 for coronary, n = 432 for aorta, n = 663 for tibial artery). Mean R-values (R_m_) across arteries for all transcripts were computed, and R_m_ values for the cGAS-STING panel were extracted and compared to the median R_m_-value for the respective list of R_m_-values (very close to 0, dotted line). *SRF*, *MYOCD*, and *MRTFA* deviated significantly in the negative direction from the median, while *MRTFB* did not (**d**, one sample Wilcoxon test). Examples of correlations between myocardin (*MYOCD*) and transcripts from the cGAS-STING panel in human coronary artery are shown in panels (**e**–**g**, n = 240, Spearman). Additional examples from aorta and tibial artery are shown in Supplemental Fig. 1. In panel (**h**), tibial artery samples (n = 663) from GTEx were stratified, and transcripts levels were compared between those with the highest (top 10%) SRF expression and those with the lowest (bottom 10%) SRF expression using Mann–Whitney tests. Contractile transcripts are shown to the left and inflammatory transcripts are shown to the right. **p < 0.01, ****p < 0.0001.

A statistics description for Fig. 1 is provided in the “*Bioinformatic analysis*” section above. All results obtained from RT-qPCR experiments were log2-transformed prior to statistical testing. We used Shapiro–Wilk’s test for normality and either F-test or Brown–Forsythe tests for examining homogeneity of variances. The Mann Whitney test (two-group comparisons) or Kruskal–Wallis one-way ANOVA with Dunn's post hoc test (multi-group comparisons) were used for non-parametric data. For parametric data, if the homogeneity test passed, an unpaired t-test was used for two-group comparisons, and an ordinary one-way ANOVA with Tukey's multiple comparisons test for multi-group comparisons. Otherwise, two-group comparisons were tested using an unpaired t-test with Welch's correction, and multi-group comparisons were done using Welch’s one-way ANOVA test with Dunnett's T3 post hoc test. p < 0.05 was considered significant throughout.

## Results

### Bioinformatic analyses suggest repression of cGAS-STING signaling by MRTFs

We approached our hypothesis by interrogating an RNA-sequencing dataset where myocardin (MYOCD) was overexpressed in human coronary artery SMCs^8,28^. A cGAS-STING panel^17^ comprising 56 transcripts showed reduced expression upon MYOCD overexpression compared to control (null virus, one sample Wilcoxon test, Fig. 1a,b). In the work from which the cGAS-STING panel was retrieved, IRF3, the second major arm below STING besides NF-κB, was inactivated. This suggests that the 56-gene panel used primarily contains NF-κB target genes. We also treated individual transcripts as categorical variables, being either (i) reduced (adjusted p < 0.01), or (ii) unchanged or increased (Fig. 1c). 3.4-fold more cGAS-STING genes were reduced by MYOCD than expected by chance (Fig. 1c, Fisher exact test, p < 0.00001). Because MYOCD expression is reduced when SMCs are cultured in vitro, these analyses suggest that overexpression of MYOCD towards in situ levels leads to inhibition of cGAS-STING signaling.

Next, we correlated all MRTFs and *SRF* versus all other transcripts in the three arteries in the GTExPortal database (tibial artery, n = 663, aorta, n = 432, coronary artery, n = 240). Mean R-values (R_m_) for all transcripts across arteries were computed and the resulting list was interrogated with the panel of cGAS-STING target genes. The R_m_-values for the panel were extracted and compared to the R_m_-median for all correlations. *SRF*, *MYOCD*, and *MRTFA* correlated more negatively with cGAS-STING target genes than expected by chance, while *MRTFB* did not (Fig. 1d). The overall analysis in Fig. 1d was based on 674,400 individual correlation analyses, and artery-segregated analyses yielded essentially similar results (Supplemental Fig. 1a–c). Exemplary correlations between MYOCD and individual cGAS-STING target genes (*AREG*, *CXCL3* and *PLAT*) in human coronary artery are shown in Fig. 1e–g (Spearman), and six additional examples of individual correlations in tibial artery and aorta are shown in Supplemental Fig. 1d and e. In another approach, we stratified tibial artery samples based on *SRF* expression and compared levels of transcripts representative for the SMC contractile phenotype (Fig. 1h, left) and inflammatory transcripts (Fig. 1h, right) between the extremes (top 10% vs. bottom 10%). Classical SMC marker genes were lower in the group with low *SRF* expression compared to the group with high *SRF* expression, while inflammatory transcripts from the cGAS-STING panel differed in the opposite direction. Hence, transcriptomic associations in human arteries argue that *MYOCD*, *MRTFA*, and *SRF*, but not *MRTFB*, restrain inflammation in the human arterial wall.

### MRTFs suppress inflammatory transcripts in bronchial SMCs

**Figure 2:**
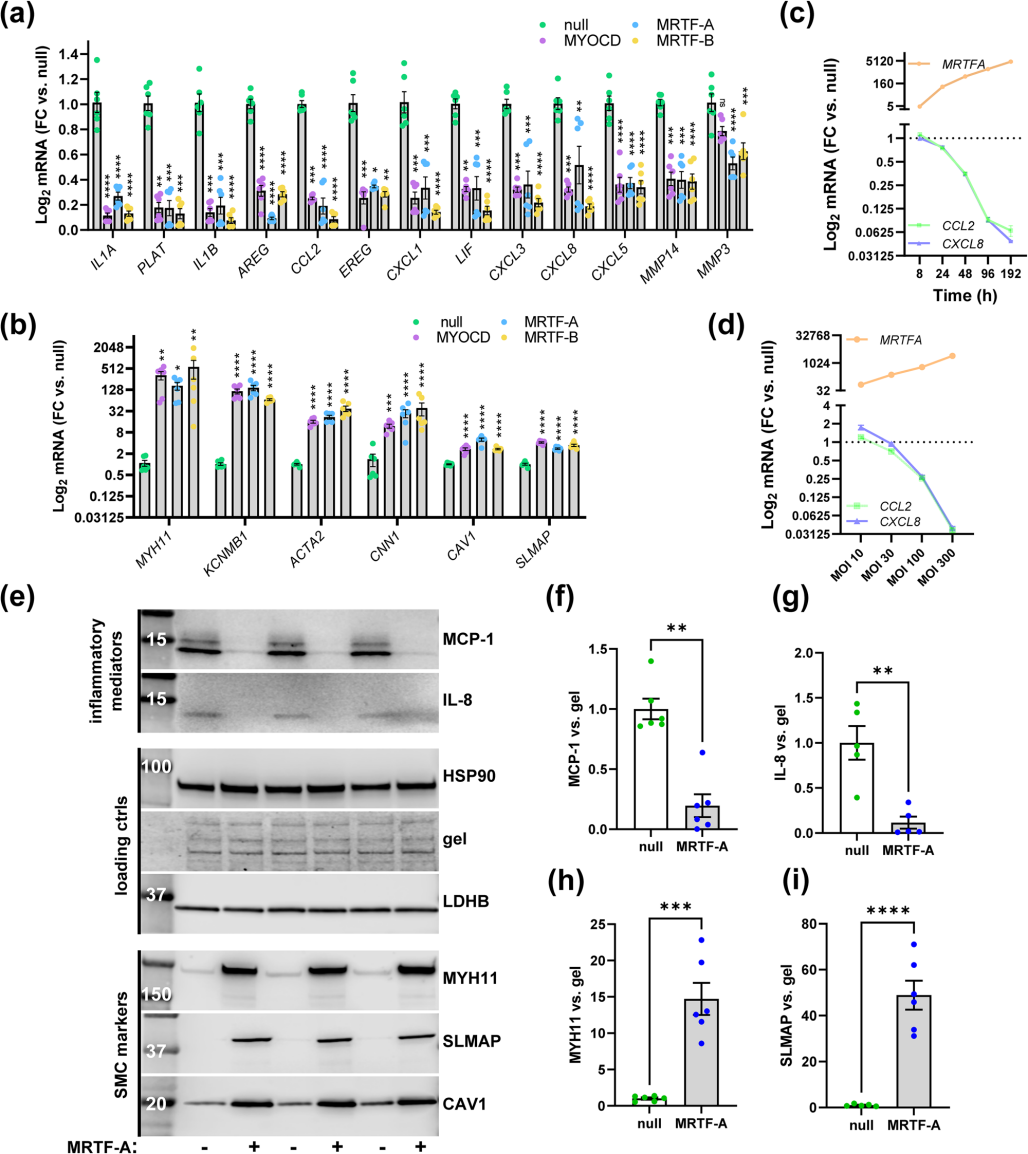
Suppression of cGAS-STING targets by MRTFs in human bronchial SMCs. Human bronchial SMCs were transduced with adenoviruses for overexpression of MYOCD, MRTF-A, and MRTF-B. Null virus was used as control. After cell harvest at 96 h and RNA isolation, pro-inflammatory mediators (**a**) and contractile markers (**b**) were assayed by RT-qPCR (one-way ANOVA with Dunn's post hoc test, n = 6 for all). In panel (**c**), Ad-CMV-MRTFA or Ad-CMV-Null virus was added at time 0 h, and cells were harvested for RNA isolation at different times. *CCL2*, *CXCL8*, and *MRTFA* were assayed using RT-qPCR. At 8 h, *MRTFA* had increased fivefold, but *CCL2* and *CXCL8* remained unchanged. At 192 h *MRTFA* had increased 4500-fold, and *CCL2* and *CXCL8* were reduced by > 90% (n = 3). Panel (**d**) shows dose–response data where MRTF-A virus was added at different concentrations (MOI = multiplicity of infection). Graded suppression of *CCL2* and *CXCL8* was seen at the mRNA level as *MRTFA* increased (n = 3). (**e**) Human bronchial SMCs were transduced with null and MRTF-A virus for 8 days, and protein lysates were prepared for western blotting. We assayed MCP-1 (*CCL2*) and IL-8 (*CXCL8*) as examples of pro-inflammatory mediators, and MYH11, SLMAP, and CAV1 as examples contractile (SMC) markers. The same amount of protein was loaded in all lanes on the gel, and we used HSP90 and LDHB as loading controls. As an additional loading control, we also stained the gel after transfer (gel). Levels of MCP-1, IL-8, MYH11, and SLMAP were normalized to proteins remaining on the gel and analyzed using Mann Whitney (**f**, **g**) or unpaired t-tests (**h**, **i**), depending on the outcome of the normality test. *p < 0.05, **p < 0.01, ***p < 0.001, ****p < 0.0001.

In addition to arteries, STING is expressed in the lung. In fact, rare gain-of-function mutations in STING underlie a severe autoinflammatory disease called SAVI, or STING-associated vasculopathy of infancy, and children with SAVI suffer from combined vasculopathy and lung inflammation^33^. This may be because STING is highly expressed in structural cells, such as SMCs, of both blood vessels and airways. We overexpressed MRTFs (MYOCD, MRTF-A, and MRTF-B) in human bronchial SMCs and examined cGAS-STING target genes by RT-qPCR. Overexpression was effective in bronchial SMCs, and MRTF transcript levels increased 650–4200-fold (*MYOCD*: 653 ± 119, *MRTFA*: 4120 ± 1220, MRTFB: 4200 ± 620). Importantly, all MRTFs reduced the assayed inflammatory transcripts by well over 50% in bronchial SMCs (Fig. 2a).

MRTFs are best known for their ability to promote contractile differentiation in vascular SMCs. To ascertain that MRTFs had this effect also in bronchial SMCs, we assayed a handful of contractile markers by RT-qPCR (Fig. 2b) in the same samples used to assess inflammatory transcripts. The transcripts assayed (*MYH11*, *KCNMB1*, *ACTA2*, *CNN1*, *CAV1*, and *SLMAP*) were previously reported to be positively regulated by one or several of the MRTFs, and they are considered markers of the contractile SMC phenotype^8,31,34–37^. SMC markers were uniformly increased, and no critical differences in efficacy between the MRTFs were noted (Fig. 2b).

Time-curve experiments showed that *CCL2* and *CXCL8* were reduced after MRTF-A had started to increase and not immediately upon transduction (at 8 h, Fig. 2c), and dose–response curves showed graded repression of *CCL2* and *CXCL8* with increasing levels of MRTF-A (Fig. 2d). These experiments therefore demonstrate temporality and a graded response for the anti-inflammatory effect of MRTF-A in bronchial SMCs. Given that the effect of MRTF-A was greatest with the longest transduction time (Fig. 2c), we used 8-day transductions as standard in the remainder of our experiments.

To support a reciprocal relationship between contractile SMC differentiation and inflammation at the protein level, we assayed MCP-1 (*CCL2*) and IL-8 (*CXCL8*) alongside contractile SMC markers using western blotting (Fig. 2e). HSP90, LDHB, and proteins remaining on the gel after transfer, were used as loading controls. MCP-1 and IL-8 were reduced relative to proteins on the gel (Fig. 2e–g), while contractile SMC markers were increased (Fig. 2e,h,i). Altogether, these findings demonstrate that MRTFs regulate inflammation and contractile differentiation in human bronchial SMCs in opposite directions.

### MRTF-A silencing promotes SMC inflammation

**Figure 3:**
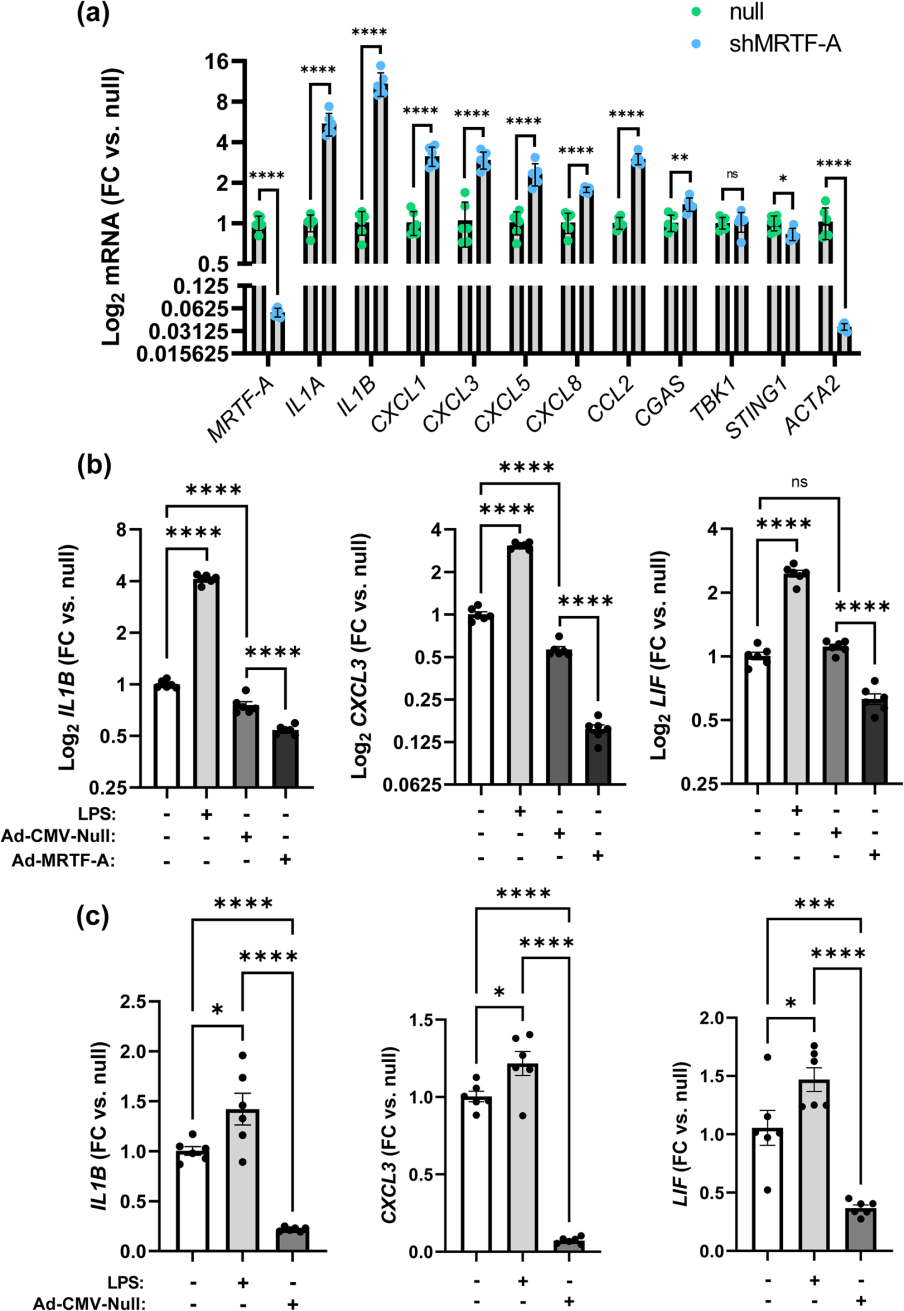
MRTF-A silencing increases pro-inflammatory mediators in human bronchial SMCs. (**a**) Endogenous MRTF-A was silenced in human bronchial SMCs using a short hairpin followed by quantification of inflammatory transcripts and *ACTA2* (SMC marker) by RT-qPCR. (**b**) Human coronary SMCs were incubated with or without, null virus, MRTF-A virus, and lipopolysaccharide (LPS, 500 ng/ml), which was included as positive control. *IL1B*, *CXCL3*, and *LIF* were assayed by RT-qPCR. Panel (**c**) confirms the anti-inflammatory effect of null virus in human bronchial SMCs. *p < 0.05, **p < 0.01, ***p < 0.001, ****p < 0.0001.

To examine if endogenous MRTF-A exerts an anti-inflammatory effect, we next silenced MRTF-A in bronchial SMCs and examined seven of the cGAS-STING driven pro-inflammatory transcripts. Knockdown of MRTF-A increased *IL1B*, *IL1A*, *CXCL1*, *CXCL3*, *CXCL5*, *CXCL8* and *CCL2* arguing that endogenous MRTF-A dampens inflammation in airway SMCs (Fig. 3a). We also assayed *CGAS*, *TBK1*, and *STING1* in this experiment, demonstrating that *CGAS* was modestly increased, whereas *STING1* was slightly reduced, and *TBK1* was unchanged (Fig. 3a). Importantly, a sizeable reduction of the contractile marker *ACTA2* occurred after MRTF-A silencing (Fig. 3a), supporting a reciprocal relationship between inflammatory mediators and contractile SMC markers also in the setting of MRTF-A silencing.

Because we were concerned that the pro-inflammatory basal state of cultured SMCs may be due to use of DNA-carrying adenoviruses for overexpression and silencing, we ran a control experiment comparing expression of cGAS-STING-regulated genes between virus-free and virus-transduced (Ad-CMV-Null and Ad-MRTF-A) SMCs. Lipopolysaccharide (LPS) was included as positive control, and LPS consistently displayed a pro-inflammatory impact (Fig. 3b,c). In contrast, the effect of the viral vector depended on the proinflammatory mediator. *IL1B* (Fig. 3b, left) and *CXCL3* (Fig. 3b, middle) were modestly reduced by null vector. *LIF* on the other hand was unchanged (Fig. 3b, right). Importantly, Ad-MRTF-A was anti-inflammatory relative to null throughout (Fig. 3b). Thus, the adenovirus did not appear to act in a pro-inflammatory manner in our cultured arterial SMCs. An experiment with similar design, but not including Ad-MRTF-A, was run using bronchial SMCs to confirm the anti-inflammatory effect of null virus (Fig. 3c). In this case, null virus reduced basal inflammation somewhat more effectively, arguing that inclusion of null virus as a control is essential. Taken together these experiments show that endogenous MRTF-A limits SMC inflammation, and that the pro-inflammatory basal state of SMCs in culture is independent of adenoviral transduction.

### MRTFs suppress TBK1 phosphorylation

**Figure 4:**
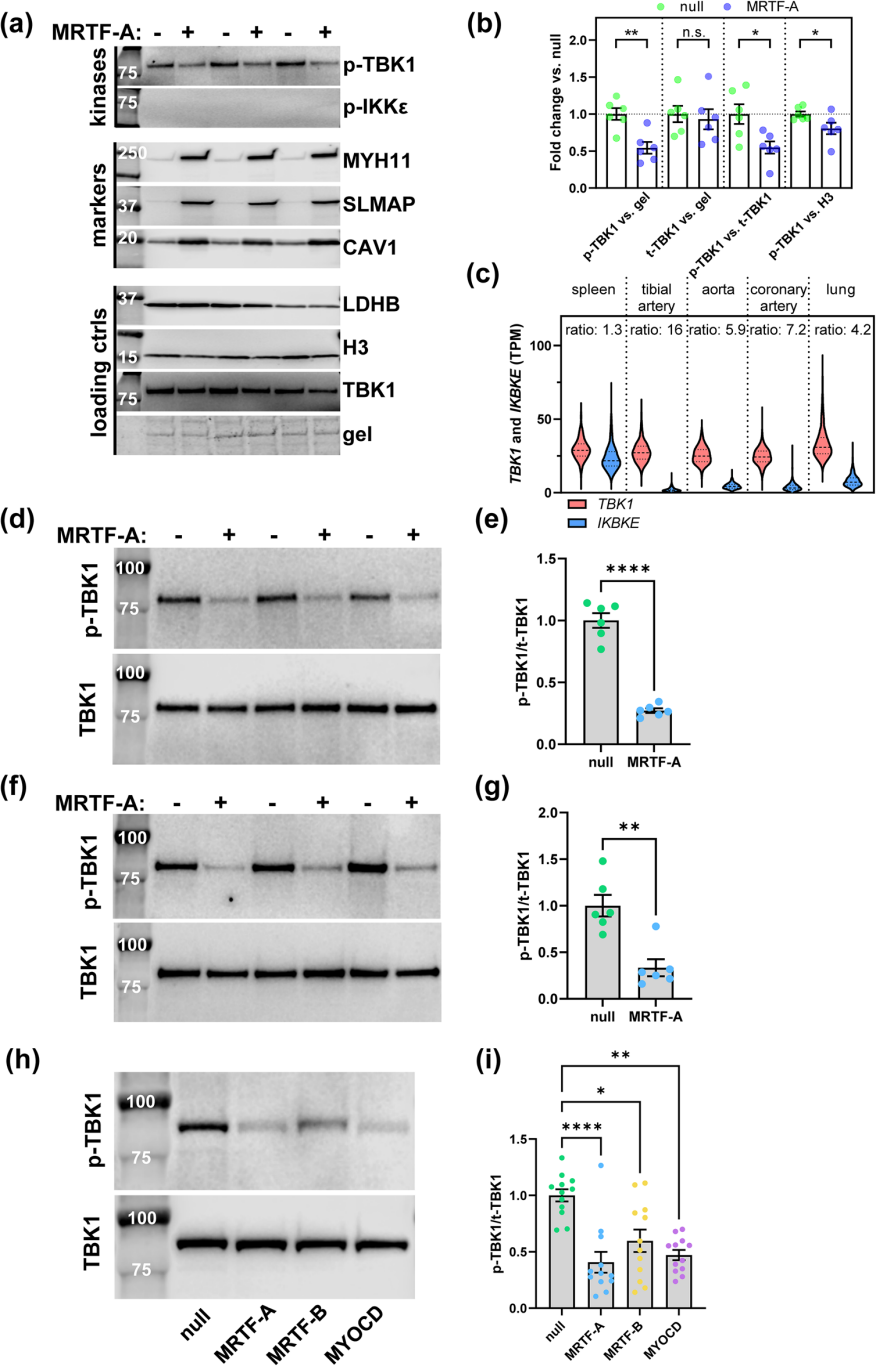
MRTFs suppress TBK1 phosphorylation in bronchial and coronary SMCs. Human bronchial SMCs were transduced with null or MRTF-A virus for 8 days. Cells were harvested, and proteins were isolated for western blotting (**a**). We assayed phosphorylation of the kinases TBK1 (p-TBK1, Ser172) and IKKε (p-IKKε), three contractile differentiation markers (markers), and four loading controls (loading ctrls) in the same lysates. Panel (**b**) shows analysis of p-TBK1 and total TBK1 versus proteins remaining on the gel (n = 6, unpaired t-tests), p-TBK1 versus t-TBK1, and p-TBK1 versus H3. Panel (**c**) shows levels of *TBK1* and *IKBKE* in spleen (n = 241), tibial artery (n = 663), aorta (n = 432), coronary artery (n = 240), and lung (n = 578) (in transcripts per million, TPM, from GTExPortal.org). The ratio of median expression (*TBK1*/*IKBKE*) was calculated for all tissues. This ratio was considerably higher in arteries and lung than in spleen. In new and independent experiments, human bronchial SMCs were next transduced with either null or MRTF-A virus and phosphorylation of TBK1 (Ser172) versus total TBK1 was assessed using western blotting (**d**). Summarized data after normalization of p-TBK1 to total TBK1 is shown in (**e**) (n = 6, unpaired t-test). Panels (**f**) and (**g**) show similar analyses using coronary artery SMCs (n = 6, Mann Whitney test). In (**h**) and (**i**), coronary SMCs were transduced in parallel with null, MRTF-A, MRTF-B, and MYOCD viruses followed by western blotting for p-TBK1/TBK1 (n = 12, Kruskal Wallis ANOVA, with Dunn’s post-hoc test). *p < 0.05, **p < 0.01, ****p < 0.0001.

Immediately upstream of NF-κB in the cGAS-STING pathway are the pro-inflammatory kinases TANK binding kinase-1 (TBK1) and I-κB kinase epsilon (IKKε) which become phosphorylated upon activation of STING. TBK1 is also known as NAK, which stands for NF-κB-activating kinase, and overexpression of TBK1/NAK induces phosphorylation and degradation of IκBα, causing nuclear translocation of RelA in the NF-κB complex^38^. To explore if the activation levels of TBK1 and IKKε were affected by MRTF-A, we first compared null and MRTF-A-transduced bronchial SMCs using western blotting (Fig. 4a). Three contractile markers (MYH11, SLMAP and CAV1), and four loading controls (total TBK1, Coomassie-stained proteins remaining on the gel, LDHB, and Histone H3) were included to assess contractile differentiation and for normalization of TBK1/IKKε phosphorylation. While phosphorylation of TBK1 (p-TBK1) was readily detectable and appeared reduced with MRTF-A, p-IKKε was undetectable (Fig. 4a, top). The contractile markers increased with MRTF-A compared to null as expected (Fig. 4a, middle). We next normalized p-TBK1 versus different loading controls and versus protein on the gel (Fig. 4b). A significant reduction of p-TBK1 was observed for normalization versus gel, versus total TBK1, and versus H3 (Fig. 4b), but total TBK1 was unchanged (Fig. 4b).

Poor detectability of p-IKKε suggested low expression. To examine this, we compared mRNA expression levels for TBK1 (*TBK1*) and IKKε (*IKBKE*) in spleen, arteries, and lung (GTExPortal data). Spleen was included as an example of a tissue rich in immune cells. *TBK1* and *IKBKE* mRNA levels were similar in spleen (the median *TBK1*: *IKBKE* ratio was 1.3, Fig. 4c) consistent with the view that these kinases act redundantly in immune cells^18^. In arteries, *TBK1* was expressed at the same level as in spleen, but *IKBKE* expression was considerably lower (*TBK1*: *IKBKE* ratio > 5.9, Fig. 4c). *IKBKE* expression was low also in lung, but the *TBK1*: *IKBKE* ratio was not quite as extreme as it was in arteries (Fig. 4c, right). These findings suggest that TBK1 may play a more dominating, and thus less redundant, role for NF-κB activation in SMCs from arteries and lung compared to immune cells due to lower expression of IKKε.

Our initial experiment (Fig. 4a) on TBK1 phosphorylation was exploratory as it included more than one outcome measure (Fig. 4b). We therefore performed new, and completely independent, experiments where the single and pre-specified outcome measurement was the p-TBK1 versus TBK1 ratio. In the new independent experiments, phosphorylation of TBK1 (versus total TBK1) was reduced by ≈70% with MRTF-A compared to null in both bronchial (Fig. 4d,e) and coronary artery (Fig. 4f,g) SMCs. We next compared the different MRTFs with null in parallel experiments and observed significant repression of TBK1 phosphorylation with all of them in coronary artery SMCs (Fig. 4h,i). Altogether, these findings supported the idea that the anti-inflammatory effect of MRTFs may rely on the cGAS-STING pathway.

### No prominent STING phosphorylation in cultured SMCs

**Figure 5:**
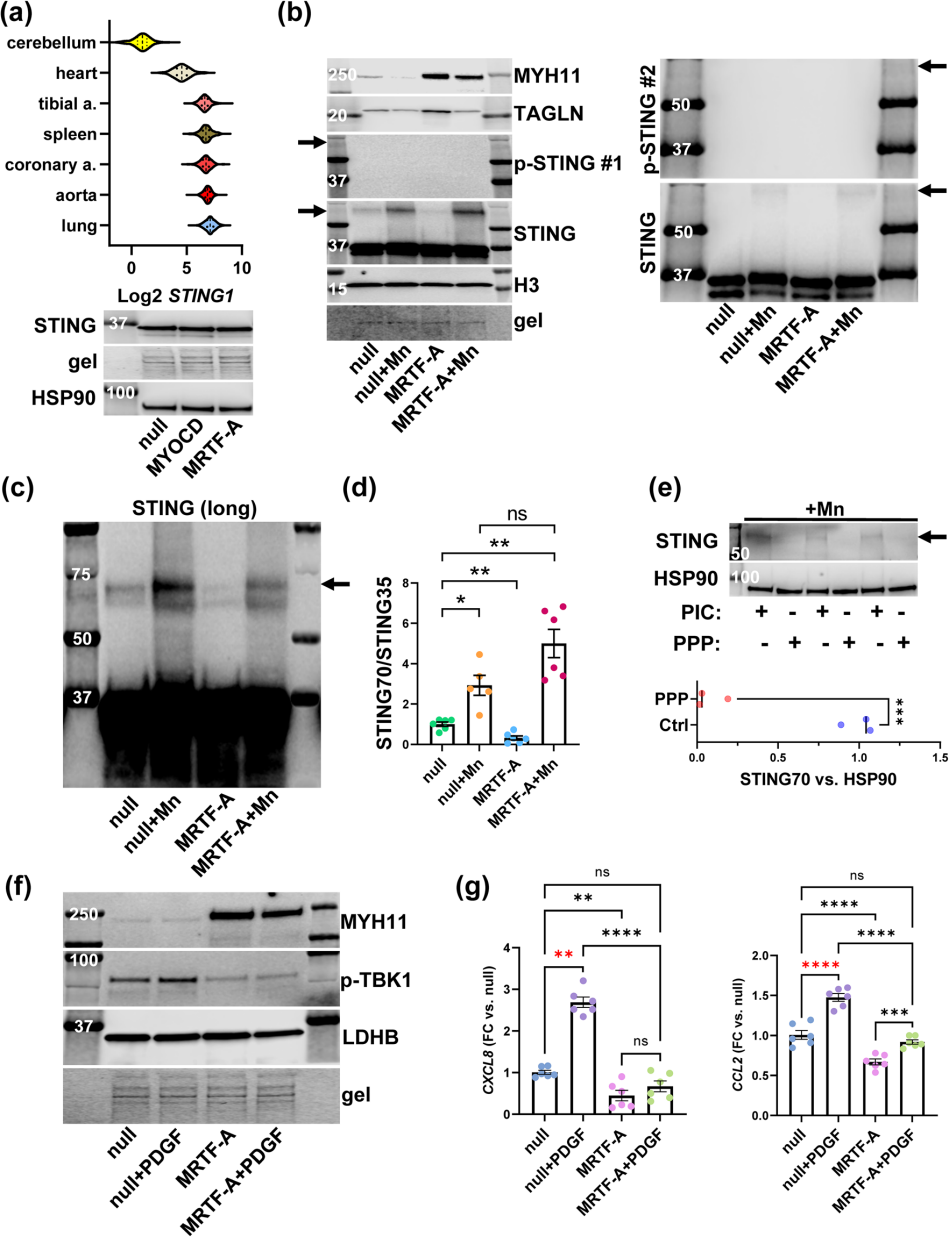
STING is highly expressed in lung and arteries, but minimally activated in cultured SMCs. Panel (**a**) shows *STING1* expression in different organs (top, from GTExPortal.org) along with a western blot for total STING in cultured coronary artery SMCs (bottom). Panel (**b**) shows blots for phospho-STING (Ser366) in lysates from cells transduced with null and MRTF-A virus and treated with MnCl_2_ (1 mM) or vehicle, respectively. Two different primary antibodies for P-STING were used (#1 and #2), and the same lysates were used to the left and to the right, so the loading controls for the blots to the left apply to those to the right. A 70 kDa STING band was detected in samples treated with MnCl_2_ (arrows) in addition to the STING doublet at 35 kDa. SM22α and MYH11 were included as markers of contractile differentiation. In panel (**c**) a longer exposure of total STING shows manganese-responsive bands migrating around 70 kDa clearly. This blot used the same lysates as in (**b**). Panel (**d**) shows summarized data where STING bands at 70 kDa were normalized to STING bands at 35 kDa (n = 5–6, one way ANOVA, Tukey’s post-hoc test using Log2-transformed data). To address if STING70 resulted from phosphorylation, lysates were incubated with phosphatase inhibitor cocktail (PIC) or protein phosphatase (PPP). PPP treatment eliminated STING70 (**e**, bottom shows summarized data, unpaired t-test). Because the (high) basal TBK1 phosphorylation in cultured SMCs appeared independent of STING Ser366 phosphorylation, we addressed if platelet-derived growth factor (PDGF), present in culture medium and released from cells, may promote TBK1 phosphorylation in panel (**f**). PDGF-BB (50 ng/ml) caused a modest increase of P-TBK1 (vs. LDHB) that was not seen in cells transduced with MRTF-A (n = 3). An experiment with similar design and larger sample size was thus run to assess the inflammatory transcripts *CXCL8* and *CCL2* by RT-qPCR (g, one-way ANOVA with Tukey’s multiple comparisons test, n = 6). PDGF increased *CXCL8* and *CCL2*, and this effect was eliminated or attenuated in cells transduced with MRTF-A. *p < 0.05, **p < 0.01, ***p< 0.001, ****p < 0.0001.

STING (*STING1*) is an ER membrane protein and upstream activator of TBK1, and STING oligomerizes upon activation. To ascertain that STING is expressed in the vascular wall and lung, we first examined GTExPortal data and saw that STING expression (*STING1*) was higher in lung, aorta, and coronary artery than it was in heart and cerebellum (Fig. 5a, top). Moreover, western blotting for STING using coronary artery SMCs revealed a prominent doublet at 35 kDa (Fig. 5a, bottom), suggesting that SMCs contribute to STING expression in the arterial wall. The STING doublet at 35 kDa was not significantly affected by either MYOCD (0.87 ± 0.05, p > 0.05, n = 3) or MRTF-A (0.89 ± 0.03, p > 0.05, n = 3) compared to null (1.0 ± 0.07, n = 3).

Because STING is phosphorylated on the formation of a complex with TBK1, we set out to determine STING activation using p-STING antibodies. We included the positive control manganese (1 mM MnCl_2_), which promotes cGAS-STING activation in a DNA-dependent manner by acting directly on the level of cGAS^39^. P-STING was not detectable using two different phospho-specific STING antibodies (Fig. 5b, #1 and #2) that have been used in prior studies to detect STING activation by western blotting^40,41^. We also failed to see an increase of p-STING on stimulation with poly I:C using one of these antibodies (Supplemental Fig. 2). Faint bands at ≈70 kDa were seen with total STING antibody in manganese-treated cells (Fig. 5b, arrows beside STING blots). Prior work indicated that such bands may arise due to phosphorylation-dependent dimerization of STING^42^. We therefore used longer exposures (Fig. 5c) and a larger set of samples to analyze the STING70/STING35 ratio and found it to be higher with manganese, and lower with MRTF-A compared to null (Fig. 5c,d). To support that STING70 arises due to phosphorylation, lysates from manganese-treated cells were treated with either phosphatase inhibitor cocktail (PIC, Fig. 5e), or active phosphatase at 37 °C (PPP, Fig. 5e). STING70 was eliminated by PPP incubation (Fig. 5e, bottom). Thus, to summarize, basal STING activation is probably low in SMCs, seeing that phosphorylation was undetectable using two distinct phospho-STING antibodies, and an effect on STING Ser366 phosphorylation upstream of TBK1 is therefore probably not required for the anti-inflammatory effect of MRTF-A. However, STING70, which probably reflects phosphorylation-dependent dimerization, changes in a manner consistent with TBK1 inhibition by MRTF-A. We consequently hypothesized that MRTFs act downstream of cGAS-STING and at the level of TBK1 to limit expression of pro-inflammatory transcripts.

It was previously reported that TBK1 is activated by platelet-derived growth factor (PDGF) via protein kinase Cε^38^ in a manner that bypasses STING. PDGF is synthesized by SMCs in culture^43^, and PDGF is present in fetal bovine serum preparations used for cell culture. This raised the possibility that high basal TBK1 activation was due to PDGF present in our media. We approached this in an experiment where cells were transduced with either null or MRTF-A virus and treated with vehicle or PDGF (50 ng/ml PDGF-BB) in serum-deprived conditions. A small increase of P-TBK1 was observed in null cells treated with PDGF (5 days, + 39 ± 9%, n = 3, P = 0.02), and this effect was absent in MRTF-A-transduced cells (Fig. 5f). Moreover, when we measured *CXCL8* and *CCL2* by RT-qPCR, we observed increases with PDGF that were eliminated or dampened by MRTF-A (Fig. 5g). This argues that growth factors present in our cell culture medium contribute to basal TBK1 activity and inflammation through pathways that are independent of STING.

### MRTF-A has an anti-inflammatory effect in SMCs stimulated with the synthetic dsRNA poly I:C

**Figure 6:**
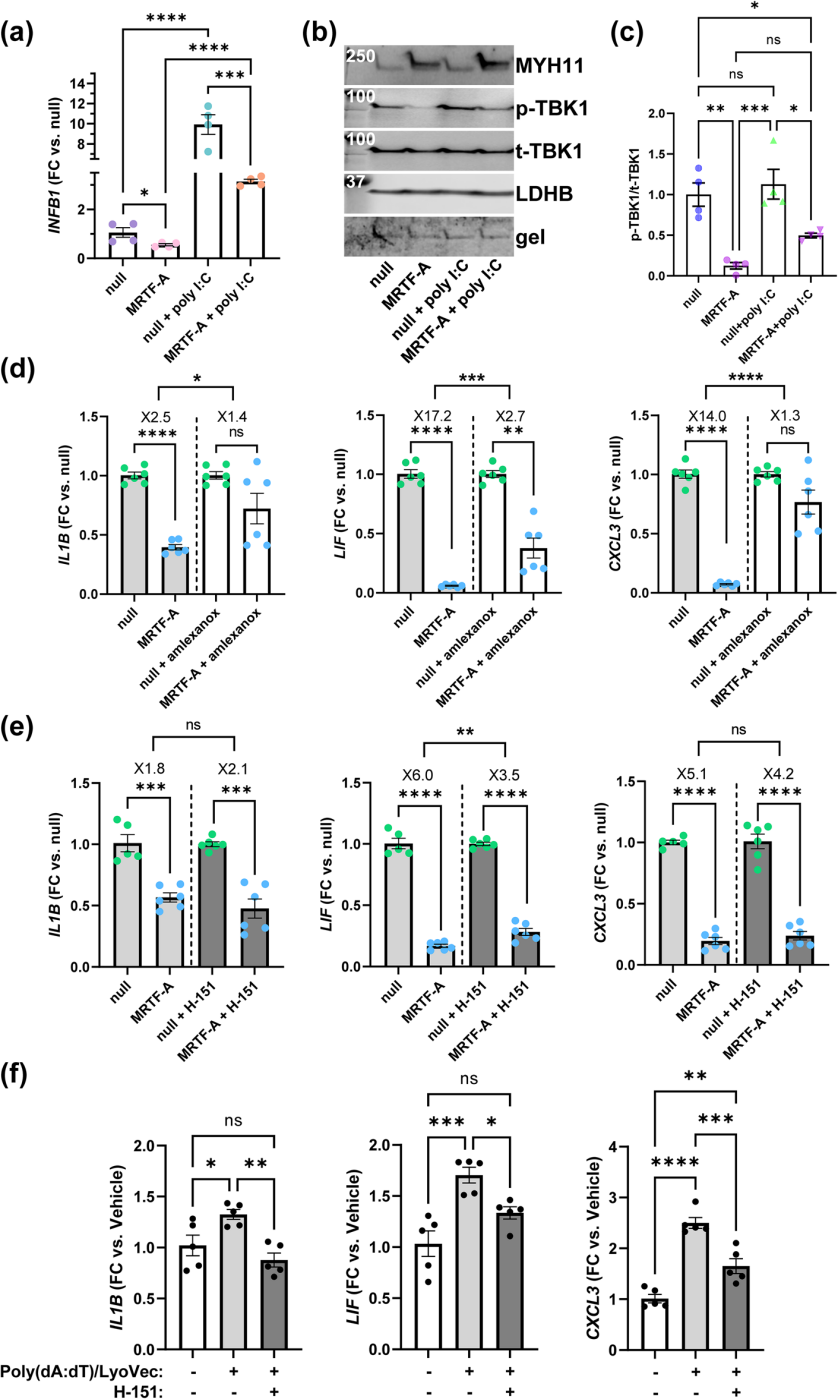
The TBK1 inhibitor amlexanox, but not the STING inhibitor H-151, reduces the anti-inflammatory effect of MRTF-A. (**a**) Cells transduced with MRTF-A or null viruses (4 days) were treated with or without poly I:C (30 μg/ml) for 24 h (**a**) or 3 h (**b**, **c**). Expression of *INFB1* (**a**) was assayed by RT-qPCR (one-way ANOVA with Tukey’s post hoc test, n = 4). Protein lysates prepared from a similar experiment after 8 days of virus transduction were assayed by western blotting (**b**). We assayed total and phosphorylated levels of TBK1 (p-TBK1, Ser172), the contractile differentiation marker MYH11, and two loading controls (LDHB and gel) in the same lanes of the same membrane. Panel (**c**) shows analysis of p-TBK1 versus total TBK1 (one-way ANOVA with Tukey’s post hoc test, n = 4). (**d**) Inflammatory suppression was compared in the presence of vehicle and amlexanox (TBK1 inhibitor, 50 µM), respectively. *IL1B*, *LIF*, and *CXCL3* were assayed by RT-qPCR. The top brackets show results from statistical testing (unpaired t-tests) of fold repression in the absence and presence of amlexanox, while subordinate brackets test for the effect of MRTF-A (unpaired t-tests or Mann Whitney tests) in the respective condition. Panel (**e**) shows an experiment with a similar design (and statistical testing) but using the STING inhibitor H-151 (3 µM). Panel (**f**) shows that treatment with synthetic dsDNA (Poly(dA:dT)/LyoVec) for 24 h, representing a positive control, increases *IL1B*, *LIF*, and *CXCL3* and that this is antagonized by H-151 (one-way ANOVA with Tukey’s multiple comparisons test throughout). H-151 in (**f**) was from the same batch, and was used at the same concentration, as in (**e**). All experiments in this figure were run using cultured human coronary artery SMCs. *p < 0.05, **p < 0.01, ***p < 0.001, ****p < 0.0001.

If MRTFs were acting directly at the level of TBK1, an effect on the interferon response would be expected, but this could not be assessed in our RNA-sequencing experiment (c.f. Fig. 1a,b) where IRF3 was likely deactivated. We thus investigated if MRTF-A suppresses interferon production using coronary artery SMCs treated with the synthetic dsRNA, poly I:C (30 μg/ml) by RT-qPCR. MRTF-A reduced basal *INFB1* expression compared to null (Fig. 6a). Poly I:C treatment (24 h) increased *INFB1* by 9.9-fold with null vector, and by only 3.1-fold with MRTF-A overexpression. Beyond demonstrating that MRTF-A has an anti-inflammatory impact after stimulation with dsRNA (Fig. 6a), this indicates that its anti-inflammatory action involves interferon suppression. We also examined TBK1 phosphorylation in similar settings (Fig. 6b), and MRTF-A reduced P-TBK1 both in presence and in absence of poly I:C, but poly I:C treatment alone had no effect on the P-TBK1 level (Fig. 6c).

### Inflammatory suppression depends on TBK1

A common denominator in all our experiments so far was that MRTFs reduce phosphorylation of TBK1. To directly test if either STING or TBK1 activity is important for the anti-inflammatory effect of MRTF-A, coronary artery SMCs were treated with the TBK1 inhibitor amlexanox (50 µM) and with the STING inhibitor H-151 (3 µM). Amlexanox significantly restrained MRTF-A-driven suppression of *IL1B*, *LIF*, and *CXCL3* (Fig. 6d). For example, MRTF-A reduced *CXCL3* by only 1.3-fold in the presence of amlexanox as compared to 14-fold in the presence of vehicle (Fig. 6d, right). This argued that the anti-inflammatory impact of MRTF-A depends critically on TBK1 activity. On the other hand, and consistent with the essentially undetectable STING phosphorylation in our culture conditions, H-151 was without effect on *IL1B* and *CXCL3* suppression, while a small but significant effect was seen on *LIF* suppression (Fig. 6e). Control experiments where we stimulated cells with dsDNA (1 μg/μl) showed that H-151, at 3 µM, suppressed the same inflammatory mediators (Fig. 6f). These experiments argued that the anti-inflammatory impact of MRTF-A depends critically on TBK1 and minimally on STING.

### MRTF-A binds TBK1

**Figure 7:**
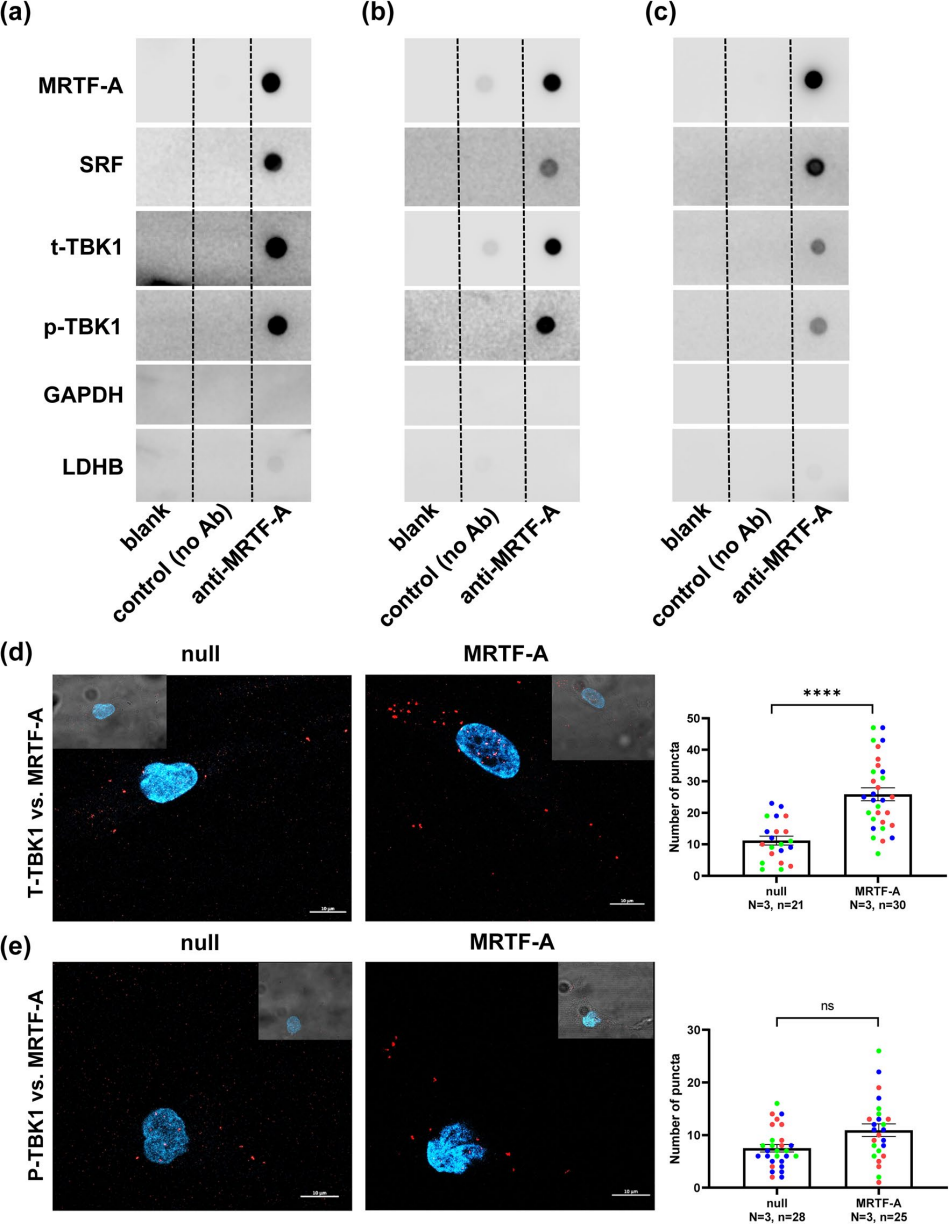
MRTF-A binds TBK1 in cell lysates and in intact cells. An antibody against MRTF-A was coupled to resin and used to isolate MRTF-A along with potential binding proteins from lysates of human coronary artery SMCs. As control, we used resin that was not conjugated to antibody. Eluates were collected after washing and proteins were assayed by dot blotting. Control eluates did not contain any of the proteins assayed for. On the other hand, MRTF-A and its prototypical binding partner SRF were present in the anti-MRTF-A eluate, as were p-TBK1 and t-TBK1. No, or very low immunoreactivity, was detected for GAPDH and LDHB. This experiment was run at both 4 (**a**) and 8 (**b**) days of MRTF-A transduction with similar results. Cells were also treated with jasplakinolide (**c**, 100 nM), which causes polymerization of actin, following MRTF-A transduction. Interaction between MRTF-A and p-TBK1/t-TBK1 was still detectable. Panels (**d**) and (**e**) show positive proximity ligation assays for TBK1 versus MRTF-A and for P-TBK1 versus MRTF-A in cells transduced with either null or MRTF-A virus. Positive puncta were seen for both antibody combinations and the number of TBK1-MRTF-A puncta increased in transduced cells. Scale bars represent 10 µm, and insets in the corners are overlays of fluorescence and brightfield images. The colors in the bar graphs represent independent experiments.

Our results so far supported the idea that suppression of cellular inflammation by MRTFs depends at least in part on TBK1. Activities of both MRTFs and of TBK1 rely on protein–protein interactions, raising the possibility MRTF-A binds to TBK1 and reduces its activity. To test for an interaction, co-immunoprecipitation (co-IP) was done. Control resin and resin conjugated with an antibody against MRTF-A were incubated with coronary artery SMC lysates. After washing and elution, MRTF-A and its canonical binding partner SRF were detectable in the eluate from resin with MRTF-A antibody, but not in the eluate from control resin (Fig. 7a). Both total and phosphorylated TBK1 were also present, whereas GAPDH and LDHB, included as negative controls, were essentially undetectable. MRTF-A-TBK1 binding was demonstrated at 4 days (Fig. 7a) and 8 days (Fig. 7b) of MRTF-A transduction. Comparable experiments for MYOCD and MRTF-B were not run because MYOCD antibodies are poor^44^, and because MRTF-B failed to show anti-inflammatory associations in human arteries (Fig. 1d). The findings with MRTF-A nonetheless argue that MRTF-A may bind TBK1.

MRTF-A is retained in the cytoplasm by unpolymerized globular actin, and actin polymerization promotes nuclear translocation of MRTF-A. To examine if actin polymerization affected the MRTF-A:TBK1 interaction, we treated cells with jasplakinolide, which causes actin polymerization, followed by co-IP. Despite an effect on cell morphology, jasplakinolide did not eradicate interaction of TBK1 with MRTF-A (Fig. 7c), but a caveat in this experiment is that our co-IP was not quantitative.

To demonstrate interaction in a cellular context, we used antibodies against MRTF-A and TBK1 and the proximity ligation assay (PLA) which detects proteins closer than 40 nm apart. In PLA fluorescent puncta indicate interaction, and such puncta were seen in the cytoplasm and in nuclei of null-transduced cells using two different antibody combinations (Fig. 7d,e, left). The number of puncta reflecting co-localization of total TBK1 and MRTF-A increased in cells transduced with MRTF-A (Fig. 7d, right). The number of puncta reflecting co-localization between phospho-TBK1, and MRTF-A did not change significantly (Fig. 7e, right). The latter finding may reflect weaker binding of MRTF-A to phospho-TBK1, or it may simply be due to reduction of phospho-TBK1 in MRTF-A-transduced cells. Nevertheless, these findings supported the view that TBK1 and MRTF-A interact inside cells, that this interaction increases with increasing level of MRTF-A, and that the interaction detected by co-IP did not arise following cell lysis.

## Discussion

The present work addresses the hypothesis that MRTFs inhibit cGAS-STING signaling to restrain inflammation in human SMCs. We demonstrate that MRTF-A binds and deactivates TBK1, a kinase downstream of cGAS-STING, offering a novel mechanism of action for MRTF-induced anti-inflammation in human SMCs. We used bioinformatic analyses along with overexpression and silencing in cultured human SMC to test our hypothesis. Bioinformatic analyses using publicly accessible RNA-sequencing datasets supported the view that myocardin suppresses a panel of cGAS-STING target genes. This association, and similar associations for *MRTFA* and *SRF*, was also seen when analyzing individual human arteries. While these associations are compelling, it is important to keep in mind that many pro-inflammatory cytokines expressed in response to cGAS-STING activity depend on NF-κB. Therefore, any mechanism that is funneled through NF-κB, including sequestration of RelA, could be involved, calling for direct testing in simplified systems. In addition to NF-κB, cGAS-STING activates IRF3, which stimulates transcription of interferons. This response rapidly desensitizes due to p38 activation and accumulation of IL1A in cell culture medium^17^. Hence, no interferon signature was apparent in our RNA-sequencing dataset under basal conditions, but using poly I:C as a stimulus, we did observe effective silencing of the *INFB1* response.

Our work demonstrates that MRTFs repress inflammation in both arterial and bronchial SMCs. The latter were chosen because STING is highly expressed in lung, in addition to arteries, and because rare gain-of-function mutations in STING give rise to vasculopathy combined with lung inflammation in a disease called SAVI^33^. All cGAS-STING target genes assayed were repressed by MRTFs in bronchial SMCs, while MRTF-A silencing increased them. Thus, specific molecular gain-of-function and loss-of function experiments support an anti-inflammatory impact of MRTFs in human SMCs from disease-relevant tissue sources. Importantly, we previously reported that inflammatory suppression by MRTFs is highly cell-type-dependent^16^, raising the possibility that the effect is conditioned by expression of TBK1 in the cGAS-STING pathway. Beyond confirming inflammatory suppression by MRTFs, our experiments on bronchial SMCs also obviate that primary SMCs in culture, unlike SMCs in healthy tissue in vivo, assume a pro-inflammatory phenotype. The basis of this transition is unclear, but it tentatively involves down-regulation of important immunosuppressive checkpoints, and growth factors present in cell culture media or released from cultured cells, such as PDGF. MYOCD itself is likely included among those checkpoints because it drops dramatically from high levels of expression in situ to low levels in cultured SMCs^45^. Use of DNA-carrying adenoviruses for overexpression and silencing is probably not involved, as shown by our control experiments comparing virus and no virus.

STING activation leads to TBK1 and IKKε activation in immune cells^18^. STING and TBK1 oligomerize at the Golgi apparatus. TBK1 is then activated by trans-autophosphorylation on S172^46,47^. In addition, STING is phosphorylated by TBK1 in the complex formed^47^. Here we show that TBK1 phosphorylation is effectively reduced in MRTF-transduced SMCs in a variety of experimental designs. However, and contrasting with our expectation, STING phosphorylation was essentially undetectable using two different phospho-STING antibodies. These observations were informative, because while implicating TBK1 in inflammatory suppression, they also argue that upstream effects are probably obsolete. Because the kinase activity of TBK1 is essential for both TBK1 and STING phosphorylation it would be puzzling if MRTFs inhibited one but not the other. We therefore used the established cGAS-STING activator, manganese^39^, and in this condition, an altered migration pattern of STING, both with manganese and following MRTF-A transduction, could be demonstrated. A note of caution is warranted regarding the use of manganese because prior studies demonstrated that it is a potent activator of integrins^48^. Nonetheless, altered STING migration depended on phosphorylation as demonstrated by phosphatase treatment. Importantly, pharmacological TBK1 inhibition blunted the immunosuppressive effect of MRTF-A, while STING inhibition had a much smaller effect. Therefore, taken together, our experiments implicate TBK1, but not STING, inhibition as contributing to inflammatory suppression by MRTFs. We also find modest activation of TBK1 by the growth factor PDGF, present in our cell culture medium and released from cultured SMCs, partly explaining the high STING-independent TBK1 activity in cultured SMCs.

The interactome of TBK1 is large^49^ and growing, and this has led to the proposition that the kinase is activated within distinct molecular complexes in different situations. Among the direct binding partners are the transcriptional co-activators YAP and TAZ, and this interaction is inhibitory^22^. Here we show for the first time that MRTF-A, which often acts together with YAP and TAZ^50,51^, also interacts with TBK1. This association appeared as robust as that with SRF which is the canonical binding partner, and it was seen both in cell lysates and inside cells using the PLA assay. It is therefore possible that MRTFs interfere with TBK1 complexes that are important for inflammation by binding and sequestering TBK1. Based on our PLA assay we propose that this interaction takes place in the cytoplasm, but we do not rule out some interaction also in nuclei. Treatment of cells with the drug jasplakinolide, which favors nuclear translocation of MRTF-A via actin polymerization, did not eliminate interaction between MRTF-A and TBK1. It would therefore appear that the overall anti-inflammatory effect of MRTF-A is localization-independent, a situation arising in part because MRTF-A inhibits pro-inflammatory mediators in the cytoplasm (TBK1) and inside nuclei (RelA and possibly TBK1). This may render MRTF-A a constitutively active suppressor of inflammation. This does not rule out indirect regulation via changes in MRTF expression. Increased MRTF expression, such as reported for Wnt stimulation^52^, or reduced expression, as reported for SMC phenotypic modulation in atherosclerosis^14^, is thus expected to have inflammatory consequences. Such indirect regulation is an interesting topic for future research.

While the current work confirms and extends previous studies from several groups showing an anti-inflammatory impact of MRTFs^12–16^, some previous studies supported a pro-inflammatory influence of MRTF-A^53–56^. For example, Yu et al.^54^ reported that MRTF-A promotes expression of six pro-inflammatory cytokines in macrophages. The same group subsequently demonstrated reduction of LPS-induced mortality in MRTF-A-deficient mice^53^, an effect likely dictated by endothelial cells. We have previously reported that the anti-inflammatory impact varies between cell types, and while MRTF-A was anti-inflammatory in arterial SMCs, no effect or a slight pro-inflammatory effect was noted in endothelial and THP1 (monocyte) cells^16^. Differences between species and TBK1/IKKε ratios are factors that could contribute to such discrepancies, but a recent study is particularly difficult to reconcile with our current findings because they used human arterial SMCs^55^. We cannot explain the basis of this intriguing dichotomy and eagerly await further studies to resolve it. We note, however, that MYOCD, MRTF-A, and SRF demonstrate robust anti-inflammatory associations in human arterial transcriptomic data.

Taken together, the current work argues that all MRTFs limit inflammation in SMCs and that MRTF-A sequesters TBK1, reducing its phosphorylation. We cannot rule out that binding and inhibition of TBK1 may be unrelated events, but TBK1 inhibition is likely functionally relevant because a small molecule inhibitor of TBK1 dampens MRTF-driven suppression of inflammation. This novel mechanism adds to the previously defined mechanism that involves sequestration of RelA, and together these effects help explain the anti-inflammatory associations of MRTFs in human arteries. It is currently unclear which of the two mechanisms (TBK1 inhibition or RelA sequestration) plays the greater role, given that they are in the same pathway, and we cannot rule out that they are interdependent. In addition, our experiments do not rule out effects that depend on transcription, and prior work demonstrated that MRTFs regulate TAZ^57–59^, a coactivator that binds and inhibits TBK1^22^. Nonetheless, our findings are probably disease relevant because myocardin expression falls in atherosclerosis, and loss-of-function interventions show that the anti-inflammatory drive of myocardin is relevant for arterial inflammation^14^. Our present findings suggest that the anti-inflammatory influence of MRTFs extends to airway smooth muscle.

### Supplementary Information

Supplementary Figure 1.

Supplementary Figure 2.

Supplementary Information 1.

## Data Availability

The datasets analyzed for this study can be found in online repositories. The names of the repositories and accession numbers can be found below: BioSample database and accessions SAMN19277810, SAMN19277811, SAMN19277812, SAMN19277813, SAMN19277814, SAMN19277815, SAMN19277816, and SAMN19277817 (https://www.ncbi.nlm.nih.gov/biosample/19277810; https://www.ncbi.nlm.nih.gov/biosample/19277811; https://www.ncbi.nlm.nih.gov/biosample/19277812; https://www.ncbi.nlm.nih.gov/biosample/19277813; https://www.ncbi.nlm.nih.gov/biosample/19277814; https://www.ncbi.nlm.nih.gov/biosample/19277815; https://www.ncbi.nlm.nih.gov/biosample/19277816; and https://www.ncbi.nlm.nih.gov/biosample/19277817), and GTEx Portal (https://gtexportal.org/home/datasets).
