# Supplementary material for: Suppression of smooth muscle cell inflammation by myocardin-related transcription factors involves inactivation of TANK-binding kinase 1
Source: Sci Rep. 2024 Jun 10;14:13321. doi: 10.1038/s41598-024-63901-3 (PMC11164896; doi:10.1038/s41598-024-63901-3)

Figure 2e, original unprocessed blots/gels

Three gels (18 lanes/gel), A, B, and C, were run with the same samples and volumes loaded. MCP-1 and IL-8 were assayed on membranes derived from gel A and gel B, respectively. Normalization in bar graphs (see Figure 2i-2f) is to protein on gel. Stack of blots in Figure 2e shows crops from one gel, as well as one LDHB, one HSP90, one MYH11, one SLMAP, and one CAV1 membrane strip. When analyzing IL-8, there was a shadow affecting null #6 and MRTF-A #6, so this pair was not analyzed (yielding n=5 instead of n=6 for both null and MRTF-A in 2g). When loading gel C (assaying HSP90, SLMAP and CAV1), there was insufficient volume of sample null #6, so this sample was omitted and MRTF-A #6 was instead loaded in that lane (yielding n=5 instead of n=6 for SLMAP null in 2i). Shown here are captures for the membranes shown in 2e from membranes A, B, or C. No cropping from the sides was applied, but cropping from top and bottom was done to focus on the membrane strip incubated with the indicated antibody. This was necessary because different brighness and contrast settings were needed to visualize the different bands. Borders to membrane strips above/below are revealed by horizontal contours and vertical misalignments of marker lanes, but this is sometimes difficult to spot. Boxes in red indicate (approximate) cropping for the final figure in this and the following montages. Brightness and contrast settings were adjusted to enhance visibility of strip demarcations and are not identical to those in the article. The band indicated by an arrow on the gel is likely caldesmon.

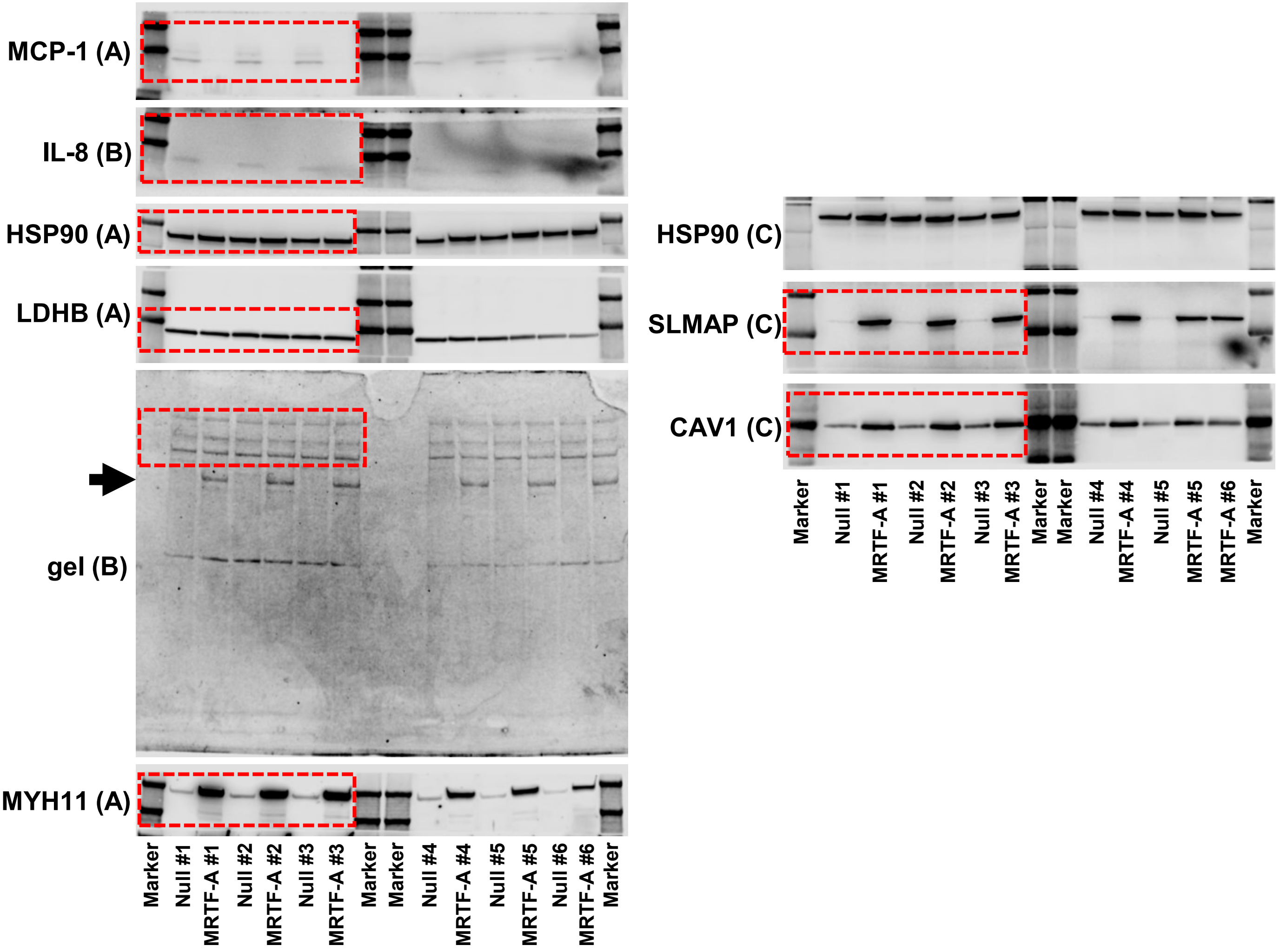

Figure 4a, original unprocessed blots/gels

The same samples (6 null and 6 MRTF-A samples) were identically loaded on two gels (A and B). Horizontal strips were cut to allow for blotting of multiple targets. As full as possible length gels and blots for crops in Figure 4a are shown here. The streak/shadow below the bands in the TBK1 blot was of no consequence for analysis. Gel A was severely torn when removed from membrane after transfer and was discarded.

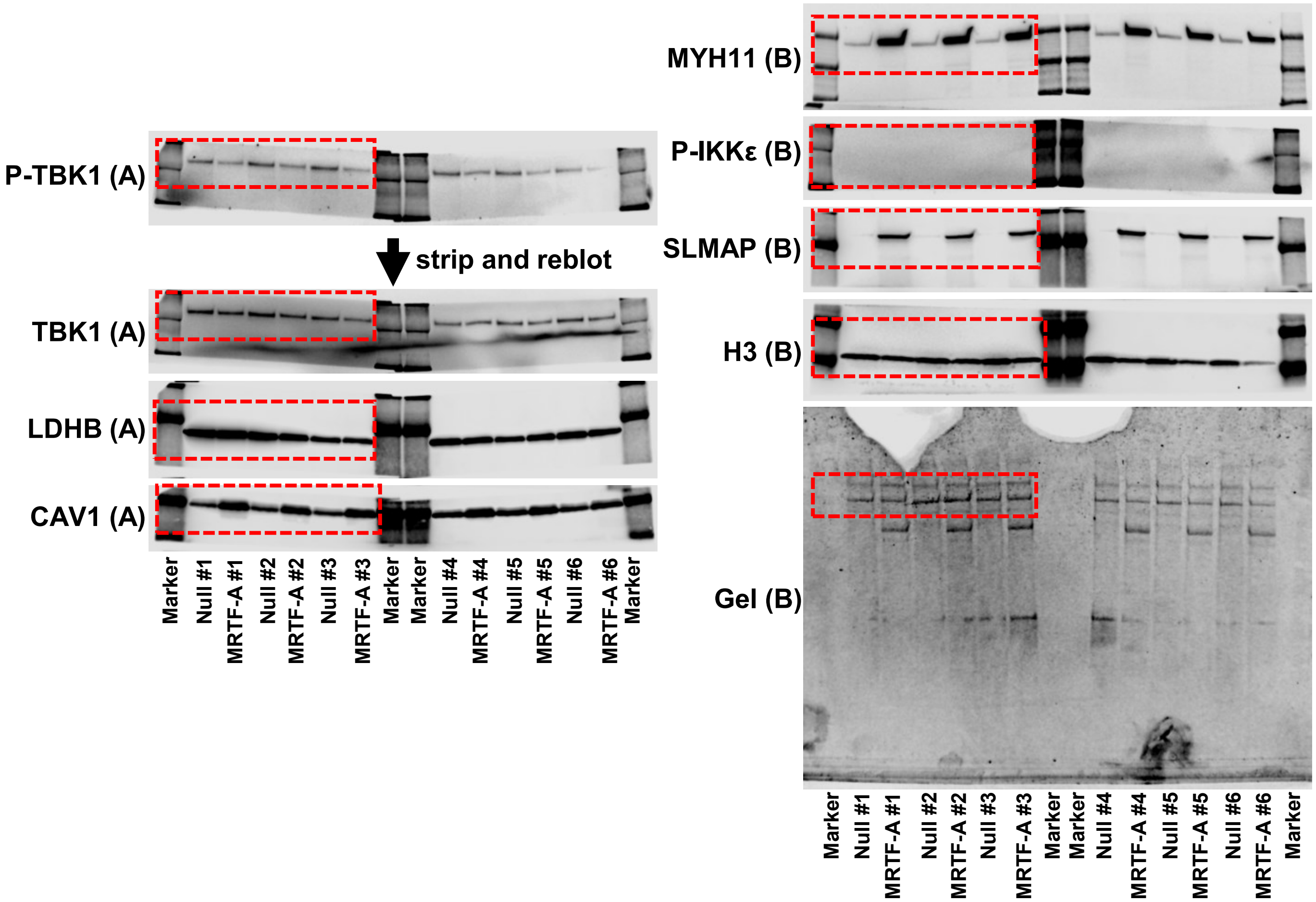

Figure 4d, original unprocessed blots

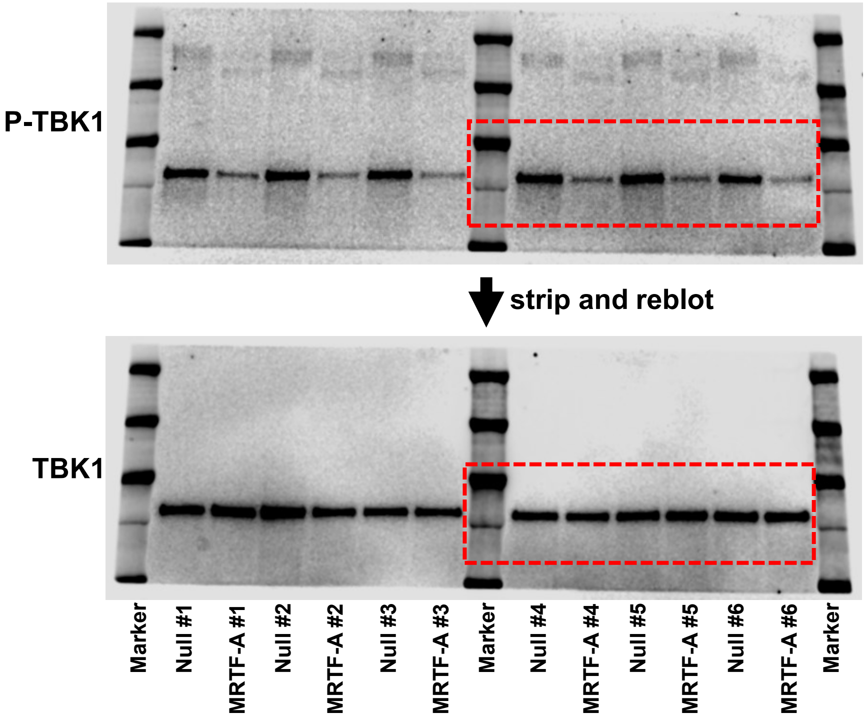

Figure 4f, original unprocessed blots

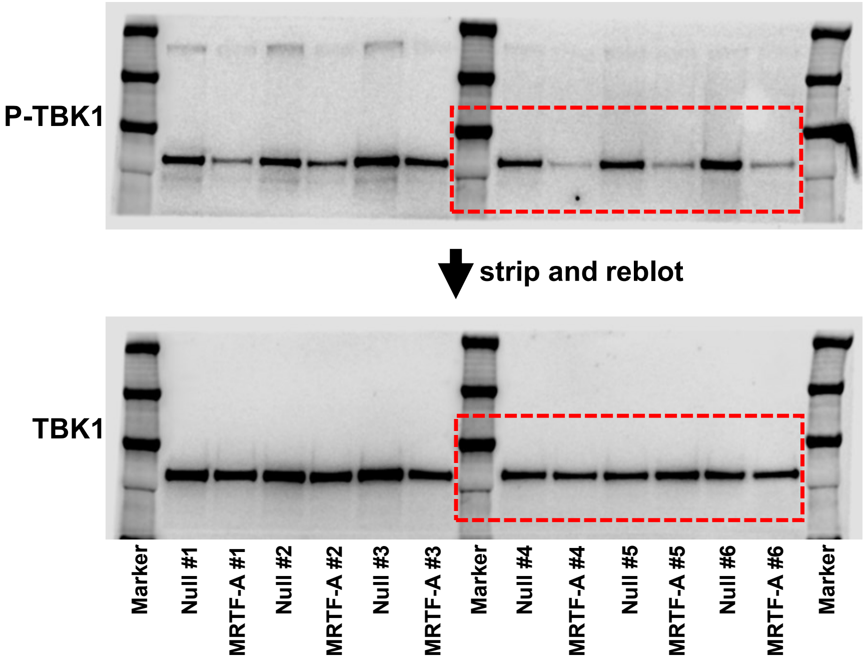

Figure 4h, original unprocessed blots

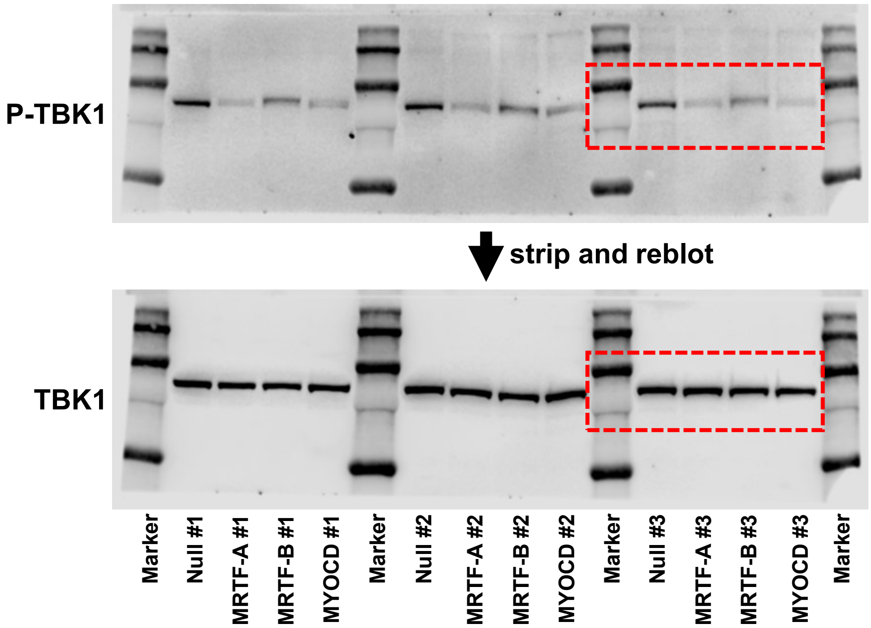

Figure 5a, original unprocessed blots/gels

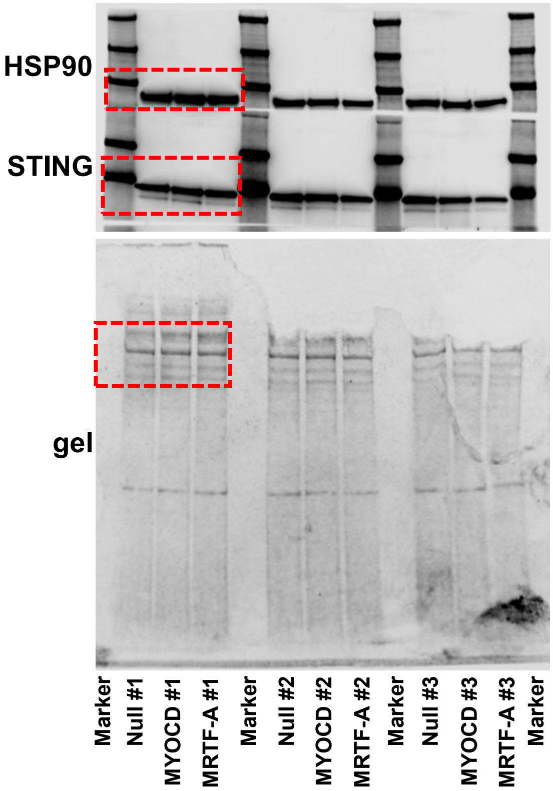

Figure 5b, original unprocessed blots/gels

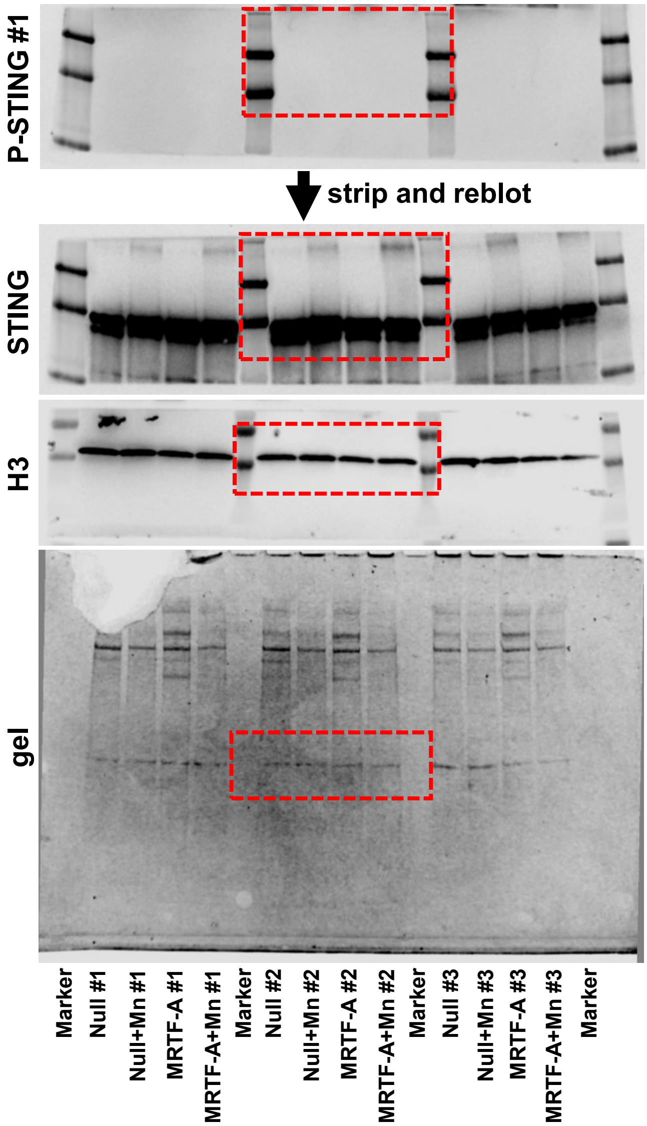

Samples (x3 in 4 groups as indicated) were loaded in identical manners on two different SDS-PAGE gels. Two different P-STING antibodies were tried, and both P-STING blots were stripped, followed by reprobing with antibody vs. total STING. Brightness and contrast settings differ from those in the article, as modifications were sometimes needed to enhance margins of the strips that were cut from the original membrane. Cutting was done to allow for blotting of multiple targets.

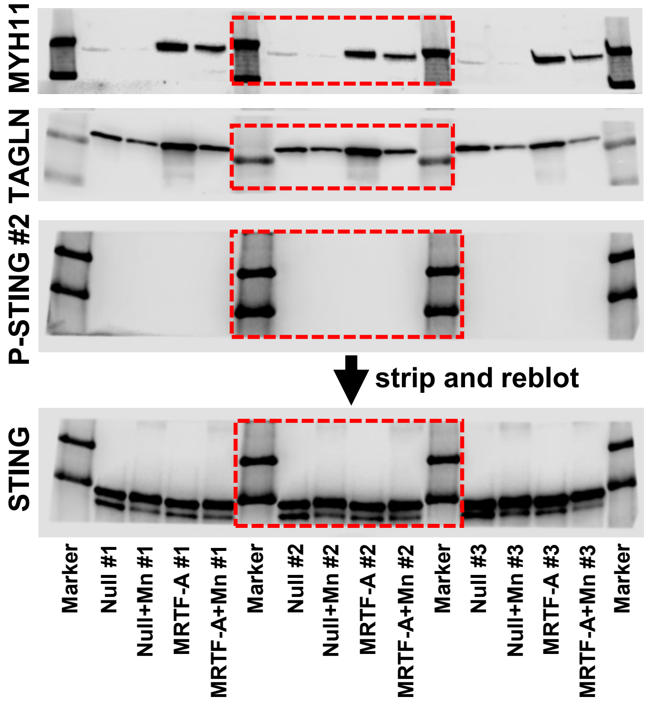

Figure 5c, original unprocessed blots

In this experiment the samples from 5b were loaded in an identical manner without cutting horizontal strips for other targets from the upper part of the membrane. Samples numbered #2 are the same as samples numbered #2 in 5b, so loading controls in 5b apply for 5c #2, but please note that STING70 was normalized to STING35 for formal analysis in 5d.

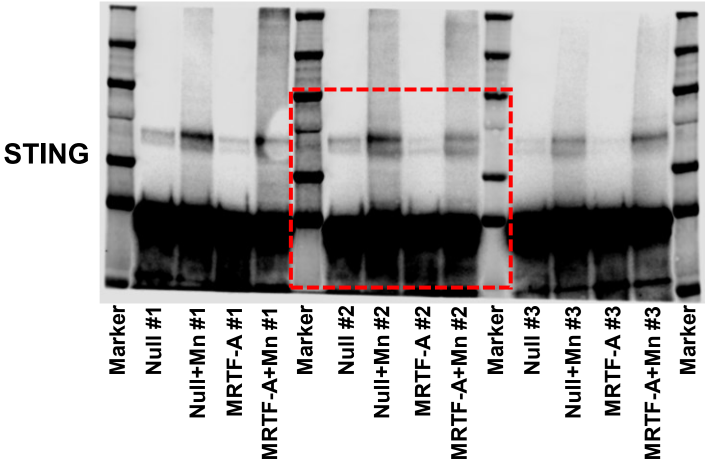

Figure 5e, original unprocessed blots

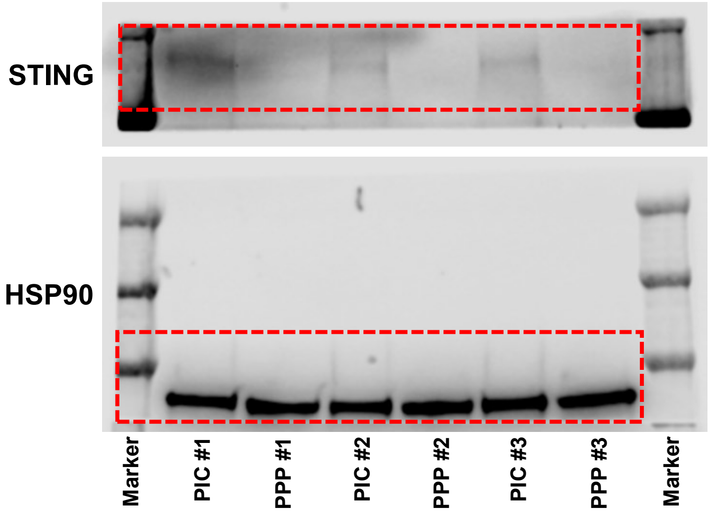

Figure 5f, original unprocessed blots/gels

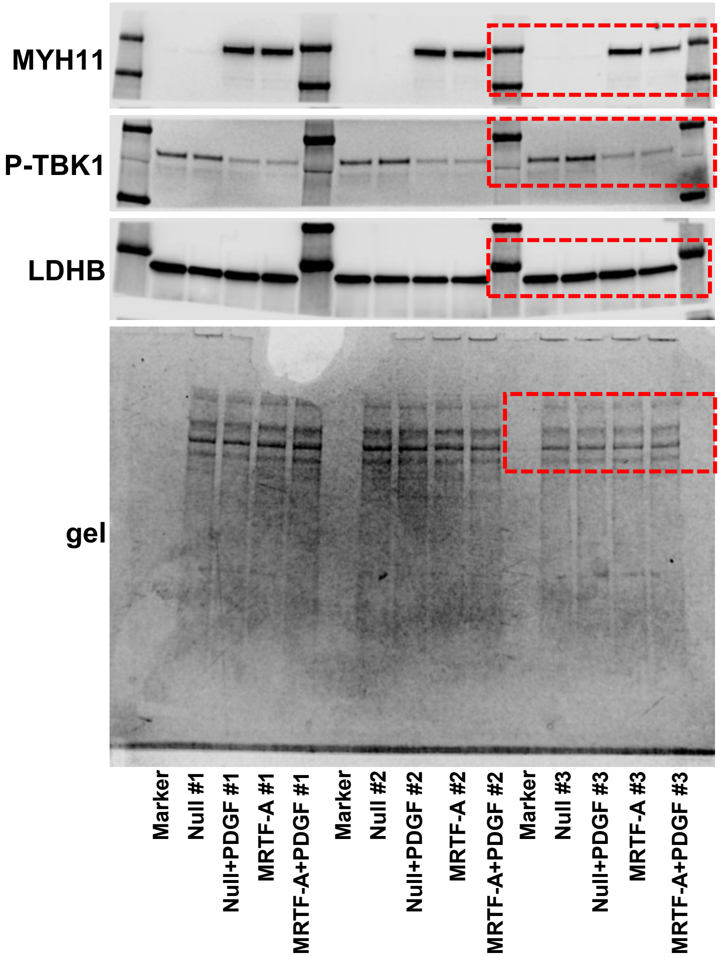

Figure 6b, original unprocessed blots

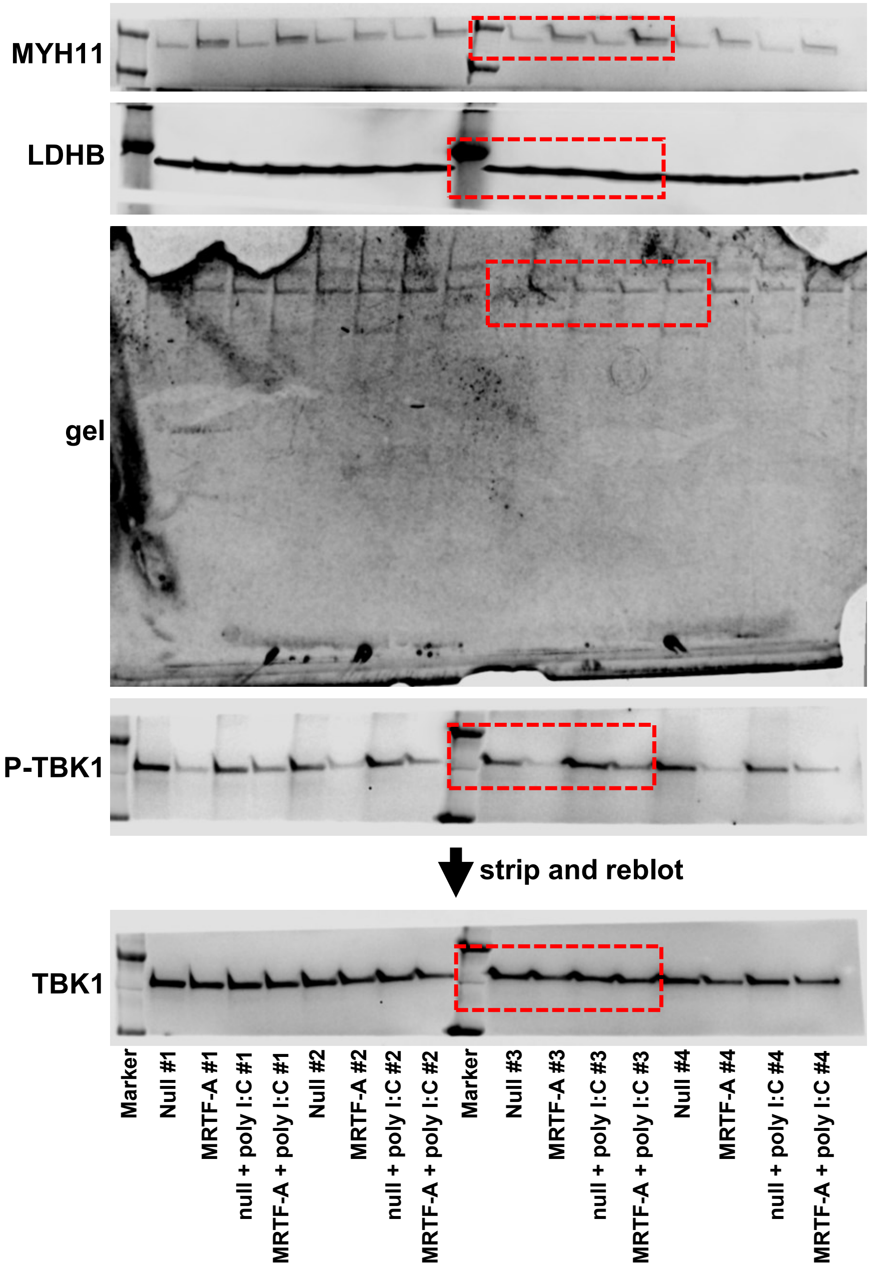

Supplement: Supplementary file 3 — Supplementary Information 1. [file 41598_2024_63901_MOESM3_ESM.pdf]
